# Real-time prediction of short-timescale fluctuations in cognitive workload

**DOI:** 10.1186/s41235-021-00289-y

**Published:** 2021-04-09

**Authors:** Udo Boehm, Dora Matzke, Matthew Gretton, Spencer Castro, Joel Cooper, Michael Skinner, David Strayer, Andrew Heathcote

**Affiliations:** 1 Department of Psychology, University of Amsterdam, PO Box 15906, 1001 NK Amsterdam, The Netherlands; 2 Department of Psychology, University of Tasmania, Sandy Bay, Australia; 3 Department of Psychology, University of Utah, Utah, USA; 4 Aerospace Division, Defence Science and Technology Group, Melbourne, Australia

**Keywords:** Cognitive workload, Detection response task, Cross-validation, Workload prediction, Human-automation teaming

## Abstract

Human operators often experience large fluctuations in cognitive workload over seconds timescales that can lead to sub-optimal performance, ranging from overload to neglect. Adaptive automation could potentially address this issue, but to do so it needs to be aware of real-time changes in operators’ spare cognitive capacity, so it can provide help in times of peak demand and take advantage of troughs to elicit operator engagement. However, it is unclear whether rapid changes in task demands are reflected in similarly rapid fluctuations in spare capacity, and if so what aspects of responses to those demands are predictive of the current level of spare capacity. We used the ISO standard detection response task (DRT) to measure cognitive workload approximately every 4 s in a demanding task requiring monitoring and refueling of a fleet of simulated unmanned aerial vehicles (UAVs). We showed that the DRT provided a valid measure that can detect differences in workload due to changes in the number of UAVs. We used cross-validation to assess whether measures related to task performance immediately preceding the DRT could predict detection performance as a proxy for cognitive workload. Although the simple occurrence of task events had weak predictive ability, composite measures that tapped operators’ situational awareness with respect to fuel levels were much more effective. We conclude that cognitive workload does vary rapidly as a function of recent task events, and that real-time predictive models of operators’ cognitive workload provide a potential avenue for automation to adapt without an ongoing need for intrusive workload measurements.

## Public significance statement

Safety and productivity can be enhanced if adaptive automation is able to predict, and so more effectively manage, the effects of fluctuations in the cognitive workload experienced by operators in high-pressure tasks. We show that it is possible to learn to predict such fluctuations based on immediate past events and actions taken by operators managing a simulated fleet of unmanned aerial vehicles.

## Introduction

Modern computer interfaces provide operators with an unprecedented level of information and control over local and remote systems. When teamed with supporting automation that can take over routine functions or provide recommendations for the operator to action, these systems have the potential to support tremendous increases in productivity. However, they also come with challenges, one of the most prominent being variations in workload. Fluctuations in workload can be associated with sub-optimal performance, which can lead to poor outcomes or complete failures of control. Underload during prolonged periods where automation takes care of all operations can cause mind wandering (Hawkins et al., 2015), sometimes called “automation neglect,” leaving the operator ill prepared for emergencies (Quandt, 2017). Although automation can help to avoid overload, it lacks the situational awareness with respect to operators levels of engagement that is often shared by human teams (Strayer et al., 2017), so can make recommendations at times that strain the operator’s cognitive capacity and even create unnecessary failures by intruding at critical times with low-priority tasks. Thus, there has been great interest over the last half century in methods to monitor operators’ cognitive engagement and workload in real time in order to enhance human-autonomy teaming through “adaptive automation” systems that better manage workload fluctuations (e.g., Groll-Knapp, 1971; Defayolle et al., 1971; Gomer, 1981; Sem-Jacobson, 1981; Humphrey and Kramer, 1994; Pope et al., 1995; Byrne and Parasuraman, 1996; Prinzel et al., 2000, 2003; Aricò et al., 2016).

However, there are many barriers to effectively deploying adaptive automation beyond the laboratory. Even if measures are available that are sensitive to relevant aspects of cognitive workload, systems based on continually monitoring workload may not be accepted by users as they are obtrusive, either because they require responses that are not relevant to the primary task or equipment attached to the body that is uncomfortable or inconvenient. Here we explore a novel approach that has the potential to avoid these issues, short-term *prediction* (i.e., over a scale of several seconds) of cognitive workload based on measures related to the state of the primary task, and primary task performance. Although predictive approaches have been used in related domains, these have been limited at longer timescales (i.e., minutes) because they have relied on blocked designs [e.g., to anticipate the effects of sleep deprivation and chronic sleep restriction on alertness and cognitive performance; Rajdev et al. (2013)]. Moreover, we are not aware of any previous research using demonstrably valid workload measures with the required temporal resolution for the short-term prediction of workload fluctuations. The development of such an approach is particularly relevant for operators of complex primary tasks where adaptive automation is needed to mitigate relatively rapid workload fluctuations.

Perhaps one reason this direction has not been explored previously is that research related to adaptive automation has mainly focused on validating retrospective workload measures. Typical studies (see Wickens et al., 2013, Chapter 11) have assessed the ability of a given measure to accurately classify performance over some period in the immediate past as coming from one of a small set of conditions where workload differs for extended periods of time (e.g., blocks of time performing different primary tasks with disparate workload demands). Although suited to validating measures, such designs are of limited relevance to scenarios in which workload fluctuates within the same task. Retrospective evaluation is also of limited use to an adaptive system that needs to anticipate prospective workload in order to take timely compensatory actions. Whether these limitations can be overcome depends not only on methodological innovations but also on the answer to a fundamental theoretical question: Do fast fluctuations in task demands lead to correspondingly fast fluctuations in spare cognitive capacity or does capacity vary as a function of the average workload over a much longer timescale. To answer this question, we assessed whether events and behavior in the last few seconds are predictive of the current level of cognitive workload. To the degree this is true, it supports the potential of real-world applications of adaptive automation.

The methodology we propose here to answer these questions differs from previous approaches in that it focuses on a single but complex primary task where different task events, and the effects of the past actions of the operator, could potentially be associated with peaks and troughs in cognitive workload. It then requires a gold standard for measuring current cognitive workload on a fast enough timescale to capture these fluctuations. For this purpose, we use the detection response task (DRT), which is recommended by the International Standards Organization (ISO) (ISO 17488, 2016) for measuring the effects of cognitive workload in driving (Bengler et al., 2012; Bruyas and Dumont, 2012; Harbluk et al., 2012). The ISO DRT procedure involves presenting a simple stimulus (e.g., a light or vibrating buzzer) with a inter-stimulus interval that varies randomly and uniformly between 3 and 5 s and requiring participants to respond with a button press when they detect the stimulus. As the workload imposed by the primary task increases, both the reaction time (RT) to the DRT stimulus and the likelihood of failing to respond (i.e., omissions) increase (e.g., Strayer et al., 2015, 2016, 2017). The DRT is an example of a secondary-task methodology, an approach which has the longest history of any type of workload measurement (e.g., Welch, 1898; Welford, 1968). The secondary-task approach has been central to the development of psychological theories of attention (e.g., Posner and Boies, 1971) and derives its theoretical basis as a workload measure from the idea of a limit on shared cognitive operations that different types of tasks depend on. The limitation has chiefly been conceptualized in two way: in terms a finite pool of central processing capacity (Welford, 1958; Kahnamen, 1973), which can cause reduced performance when shared between tasks, or in terms of access to a central processor (Posner, 1978; Welford, 1981), which can cause reduced performance through a bottleneck created because only one task can be serviced at a time (Pashler, 1984, 1994).

In our application, the DRT provides a criterion against which to evaluate the performance of different predictive models. As workload is a multifaceted construct (Wickens, 1984) it is unlikely that any one measure can capture all aspects of the operator’s experience of it (Gopher and Donchin, 1986). There is an extensive literature on measuring cognitive workload that provides a plethora of workload measures that likely tap different aspects of this construct that can be relevant to automation. In the next section, we briefly review the different measures that have been proposed with two aims; motivating our use of the DRT and providing a context for later considering our methodology might use different measures. Our proposed methodology is not necessarily dependent on the DRT. It requires only that a measure or perhaps combination of measures provide a valid and sufficiently fine-grained workload assessment (both temporally and in terms of its level) that is relevant to aspects of the primary task which might be aided by automation. Further, the measure(s) need not be entirely unobtrusive as long as primary task performance does not become unrepresentative of real-world operating conditions. This is because workload monitoring is only needed in the initial training phase required to develop predictive models. As our aim here is simply to determine whether the approach is viable in theory through the examination of one particular case (albeit one which we hope is relevant to a range of applications), we only explore the initial training phase with the DRT providing our single measure of workload.

After the review of cognitive workload measures, we report the results of an experiment in which participants managed a team of simulated unmanned aerial vehicles (UAVs) whose fuel levels constantly decreased. Participants could sample the fuel level of each UAV by hovering a mouse cursor over it and then could click to initiate refueling if that was deemed necessary. Similar multiple asset-monitoring tasks are presently of great applied interest as operators are increasingly being asked, with the aid of automation, to manage multiple agents (e.g., guiding and maintaining fleets of surveillance vehicles). Our analysis first focused on the predictive ability of the occurrence of immediately preceding task events and then explored whether improvements in prediction could be obtained using measures of the likely difficulty of the task in the next few seconds and measures of the operator’s situational awareness with respect to achieving the goals of the task. Our assessment of these measures used cross-validation techniques that develop predictive models based on a subset of participants and then test their performance with other participants (i.e., “out-of-sample” prediction). It showed that prediction, although difficult, is possible. Having established—to our knowledge for the first time—that it is possible to predict short-timescale fluctuations in cognitive workload, we finish by discussing the implications for applications. Although the successful predictive measures we developed are specific to our task, we believe that our results and methodology can be used as a basis to develop suitable predictive measures, not only for a variety of multiple-asset management tasks, but also for the many other tasks in which automation has the potential to play a beneficial role.

### Cognitive-workload measurement

In order to assess cognitive workload, prior research has typically employed some combination of primary- and secondary-task behavioral measures, physiological measures (either neurological, cardiovascular or ocular), and subjective workload assessments. These measures can be assessed against criteria such as sensitivity, selectivity, obtrusiveness, bandwidth (i.e., temporal resolution), and reliability (Wickens et al., 2013).

Primary task measures assume that performance on the operator’s main task degrades when workload increases. For example, operating a motor vehicle often worsens as cognitive workload increases either due to an increase in the complexity of the task (e.g., changes in traffic density or roadway complexity) or with the addition of a secondary task (e.g., a concurrent phone call). If there are no data limits (e.g., stimulus characteristics which affect performance), primary task measures can provide a reliable measure of workload [but see Medeiros-Ward et al. (2014)]. However, it may be difficult to identify appropriate primary-task measures and they tend to be quite specific to the task at hand. Moreover, simple primary-task measures such as the frequency of certain task events used in earlier research [e.g., changes in the number of aircraft an air traffic controller has to monitor; Ayaz et al. (2012)] often lack the necessary resolution to predict short-term fluctuations in workload. Our approach can be seen as a methodology to make such identifications in terms of measures that are predictive of workload in the near future rather than indicative of current workload. However, in order to bootstrap this process we need a workload measure that can a priori be assumed to be valid, and ideally one which might be used across a number of different primary tasks, rather than having to be developed for each new task.

Subjective approaches are perhaps the most common and easily administered type of workload assessment. Typically, they involve some sort of rating scale or scales. The NASA TLX questionnaire (Hart, 2006) is a widely used example, requiring users to retrospectively rate their subjective experience after performing a task. The ratings address six dimensions: physical and mental demands, time pressure, success, hard work, and stress. One problem with this approach for our purposes is low temporal resolution. Higher sampling frequencies are possible [e.g., Teh et al. (2014) obtained a single 10-point rating every 8 s in a driving task], but are likely to interfere with primary-task performance. A second problem is that an operator’s feelings about workload may not align with the aspects of workload that determine primary-task performance [e.g., Wickens et al. (2013) uses the example of a heuristic solution feeling easier but being less accurate than an algorithmic solution].

Physiological workload measures are a common approach in the context of adaptive automation. They involve recording biological measures, with examples including: optical imaging of cerebral blood flow (e.g., Le et al., 2018) and more general thermal imaging (e.g., Abdelrahman et al., 2017); blood pressure and electrocardiographic (ECG) recordings (particularly with respect to heart rate measures; e.g., Heine et al., 2017); eye movements and pupilometry, (e.g., Duchowski et al., 2018), as well as electrooculography (EOG), and more generally electromyography (EMG; i.e., electrical activity in muscles) recordings; electrodermal activity (EDA); e.g., Visnovcova et al., 2016); and perhaps most prominently, event-related potential (ERP) and more generally electroencephalographic (EEG) recordings (Pergher et al., 2019). Lohani et al. (2019) provide a recent review that is particularly relevant here as it also considers the practical difficulties associated with deploying each measure in the context of driving, which are frequently marked. Physiological measures are attractive because they are often continuous, or nearly so, both in time and effect magnitude. They are also often unobtrusive in the sense that they do not interfere with primary task performance, although some types may be physically cumbersome. Remote sensing offers a potential solution to the latter problem, but may be plagued by noise due to fluctuations in environmental conditions (e.g., ambient light fluctuations in pupilometry). However, continual improvements in technology have lessened many of these barriers and have also increasingly made it possible to combine different types of measures (e.g., Borghini et al., 2014; Chen et al., 2017; Liang and Lin, 2018).

Even by the 1990s, technological advances were sufficient to inspire increasing optimism about the potential of physiological measurements in adaptive automation (e.g., Byrne and Parasuraman, 1996), with Gevins et al. (1995) even suggesting that “it is reasonable to expect that in the near term a basic enabling technology will be deployed that will permit routine measurement of brain function in operational environments” (p. 169). Humphrey and Kramer (1994) first addressed an important question for real-time measurement; how much physiological data are required for accurate classification of different workload conditions? They noted that previous ERP-based studies—which typically collected 50 to 100 single trial ERPs whose average was used to discriminate among different workload conditions—could not answer this question. They developed a test based on comparing single and dual-task conditions involving an arithmetic task and a monitoring task similar to our task in which participants had to click on gauges to determine their state, and if necessary take a corrective action. Workload was assessed based on ERPs to task events (gauge sampling and the presentation of arithmetic operands), using off-line analysis to obtain various measures based on  1.3 s segments of EEG. Pools of odd and even numbered events were created and a bootstrap analysis performed on average vectors of ERP measures from one or more trials. For each of 12 participants, a linear discriminant function was estimated to classify low vs. high workload conditions based on one pool of trials then tested on the participant’s other pool of trials. Performance improved as the number of trials increased, with a cross-validation accuracy of 90% achieved between 5 and 12 trials for all participants.

These results were sufficiently encouraging to inspire a great deal of research along similar lines with EEG-based measures, including ERPs based on events associated with secondary tasks such as tone counting and oddball detection (e.g., Sirevaag et al., 1993; Kramer et al., 1995; Allison and Polich 2008). Frequency-based EEG measures, which are attractive because they do not require a link to primary- or secondary-task events, used increasingly sophisticated analysis approaches, such as individual participant multivariate analyses (e.g., Gevins et al., 1998) and analyses addressing problems with non-stationarity (e.g., Murata 2005), and were applied to a broad array of tasks and settings taking advantage of the possibility of more mobile recording (e.g., De Massari et al., 2014; Smith et al., 2001; Mijović et al., 2017). EEG has been used as a basis for adaptive automation in a closed-loop system (Pope et al., 1995; Prinzel et al., 2000) with a more recent implementation switching to ERPs because of their greater specificity to cognitive workload (Prinzel et al., 2003).

The final approach, secondary-task workload measures, requires participants to perform another task at the same time as they perform the primary task. On the assumption that the primary and secondary tasks share a limited central processing capacity or are subject to a shared bottleneck due to having components that must access a central processor, changes in primary-task cognitive workload should be associated with opposite changes in secondary-task performance. This broad theoretical basis makes the secondary-task technique potentially widely applicable, although it is important to recognize that there are cases in which tasks do not share a bottleneck or resources (Wickens 1984). A variety of secondary tasks have been used, including temporally related measures such as rhythmic tapping, interval production and time estimation, but by far the most common is the probe reaction time task (Wickens et al., 2013), of which our focus here, the DRT, is a prominent example. The DRT has has several advantages for our purposes. It is easy to implement and produces results that are easy to analyze. Further, it is sensitive to dynamic changes in workload (e.g., Strayer et al., 2017) and does not produce consequential interference on primary driving-related tasks (Castro et al., 2019; Palada et al., 2019). Strayer et al. (2015) directly compared the DRT with subjective (NASA-TLX), and physiological (ERP, time locked to the DRT stimulus) measures of cognitive workload in both simulated and on-road driving. Participants were asked to perform a number of concurrent secondary tasks that varied in difficulty. All measures varied significantly with cognitive workload; however, the degree of sensitivity differed, being marginally greater for the subjective measure (partial $\eta ^2 =.95$) than the DRT (partial $\eta ^2=.76$), both of which were substantially better than the physiological measure (partial $\eta ^2$ = .33). Given that subjective measures do not provide a sufficiently high temporal resolution, these results support the use of the DRT as our gold-standard measure of cognitive workload. As previously noted, however, our approach can be used with other workload measures, or with combinations of measures, as long as they have sufficient resolution.

In order to check the validity of the DRT in our particular application, our design included three variants of the task where difficulty, and hence average workload, differed over periods of several minutes due to the requirement to manage either 3, 5, or 7 UAVs. The increase from 3 to 5 UAVs and the increase from 5 to 7 UAVs both produced large decrements in primary task performance indicative of substantially increased workload. Hence, if the DRT is a valid measure, it should increase reliably with the number of UAVs. These conditions also provided a yardstick to calibrate the DRT so we could judge the importance of short-term workload fluctuations within a condition. If the size of the changes in DRT performance associated with short-term fluctuations is comparable to the changes in DRT performance associated with the manipulations of the number of UAVs, it will be clear that the short-term fluctuations are having a substantial impact of workload in an absolute sense.

## Experiment

We used a version of the DRT where participants responded to tactile stimulation from a buzzer using a foot peddle. The primary task required participants to monitor and refuel a fleet of UAVs. UAVs moved in straight trajectories, reflecting at screen boundaries, and were clearly visible against a homogeneous ocean background. Similar to a multiple-object tracking task, UAVs were not labeled and moved at a pace that made it easy to track a single UAV, with difficulties arising only when simultaneously following multiple UAVs. Pilot testing confirmed that the difficulty manipulation (i.e., managing 3, 5 or 7 UAVs) caused the task to vary from engaging but manageable to extremely demanding. Fuel levels, which could be monitored by hovering over a UAV with a mouse cursor, drained at a constant rate, and UAVs could only be refueled below a critical level. When a UAV ran out of fuel, it crashed and exploded and was replaced by a new UAV that appeared from the side of the screen with a high but variable fuel load and a randomly oriented trajectory. A clock was not provided on the screen so participants had to judge the passage of time subjectively in order to know when refueling might be required. Crashes could, therefore, occur because of event-based prospective memory errors (i.e., failing to ever check fuel level for a new UAV) time-based prospective memory errors (i.e., failing to refuel at the right time) and tracking errors (i.e., failing to be able to find a UAV due for refueling). Points were awarded for checking fuel levels during the critical period, and for successfully refueling, and points were subtracted for monitoring or refueling at the wrong time, and for UAVs exploding.

Participants were instructed that hovering over a UAV before its refuel window would result in a slight loss of points and doing so within the window would result in a small gain in points. Attempting to refuel a UAV outside of its refuel window would result in a larger loss of points, failing to refuel a UAV would result in the largest loss of points, and successfully refueling a UAV would result in gaining the most points. Every time the participant completed one of these actions, the number of points gained or lost would appear briefly on screen at the location of the action. No tangible reward was associated with these points, but they accumulated over the course of the blocks. Although demanding, the task was relatively simple, so participants could begin to develop expertise, as evidenced by improved performance over the course of the experiment. Measurement over a second one-hour session performed on a subsequent day allowed us to examine the moderating effects of participants developing expertise, in terms of an understanding of the points associated with different event combinations, and strategies and associated skills that enabled them to maximize points gained.

In an initial analysis, we determined participants’ sensitivity to the payoff scheme by examining how their scores, and the frequency of the five possible task events (e.g., successful or failed monitoring, successful or failed refueling, and a UAV explosion) changed from the first to the second session. We then examined the effect of the difficulty manipulation on DRT performance, with the hypothesis that clear increases in RT and omissions with increased difficulty would validate its sensitivity to cognitive workload. These analyses set the stage for our main aim, to assess whether and how we could predict real-time fluctuations in cognitive workload as measured though the proxy of DRT performance, both in terms of RT and omissions. Note that participants were explicitly instructed that, although they should respond to the DRT stimuli whenever possible, the UAV task should take priority.

We first examined whether the prior occurrence of five types of simple task events had any predictive ability for short-term fluctuations in workload, and on what timescale these task events might affect DRT monitoring (3–5 s). Our analyses also took account of potential learning and fatigue effects over the course of a session. In a second analysis, we investigated whether refined predictors, most of which aimed to index the operator’s situational awareness with respect to the fuel levels of the UAVs, were more predictive of short-term fluctuations in workload. Although the initial analysis did not reveal any strong relationships, it did provide guidance for the development of the set of refined predictors.

Because of a focus on prediction, our primary method of inference in both cases was cross-validation. This analysis differed from that of Humphrey and Kramer (1994) discussed previously in that we used less data (i.e., single vs. multiple trials) to predict a fine-grained measure of workload (i.e., predicting the continuous DRT RT measure vs. classifying discrete conditions), although we also predicted a binary omission measure. Their cross-validation was post hoc and within subject, whereas ours was more demanding, both because it involved genuine prediction (albeit over a short timescale) and was between subjects. In particular, we fit linear mixed-effect models of DRT RT and omission rates to data from five mutually exclusive groups each consisting of around 20% of the participants and the models were assessed by their ability to predict corresponding DRT performance for the left-out groups of 80% of participants. Between-subjects cross-validation is a particularly rigorous test because it is known to be more difficult than with-subjects cross-validation (e.g., Gevins et al., 1998). We assessed the stability of these predictions over participants by comparisons among results for the five “folds.” As we were interested in which predictor or group of predictors performed best, we used model selection techniques to compare a large number of models with different predictor sets.

## Methods

### Participants

Forty-six participants were recruited from a participant pool database maintained by the University of Utah. Participants were compensated $40 for completing the full study, which consisted of two one-hour sessions on different days.

### Equipment and task software

Four Windows 7 desktop computers, equipped with second-generation i7 processors with integrated graphics processors and 21-inch high-definition screens, presented the primary tracking task. In pilot testing, these systems were verified as presenting the UAV task at a steady 35 frame per second. Each computer interfaced with DRT hardware, which included a millisecond-accurate controller box running an Advanced Reduced-Instruction-Set-Computer (RISC) Machine (ARM) embedded processor and a rubberized vibrotactile motor for tactile alerting. Participants used their preferred hand to interact with the UAV task by operating a Microsoft Basic Optical Mouse connected via USB. The other hand was held at rest on the desk and used to collect Galvanic Skin Response (GSR) data. GSR readings are not analyzed in the present report.

DRT units followed the performance specifications outlined in ISO 17488, with the exception that a foot pedal switch was used to collect responses rather than the finger mounted micro-switch. Responses were recorded on a TEMCo Heavy Duty cast aluminum foot switch. A small vibration motor provided the stimuli to be detected. The motor was fastened to participants’ left shoulder using medical tape. When the motors are active, they provide a gentle vibration every 3-5 s that lasted for 1 s or until a response was made. After a recorded response, the next stimulus onset was selected randomly from a uniform distribution between 3 and 5 s. Participants were instructed to press a foot pedal switch as soon as they detected the vibration stimulus. The DRT task was active throughout the experiment. Reaction times collected from the DRT units were gathered on a micro-controller that provided sub-millisecond accuracy. Each reaction-time event was timestamped using the host computer’s clock. These timestamps were used to synchronize with data generated by the UAV task.

**Fig. 1:**
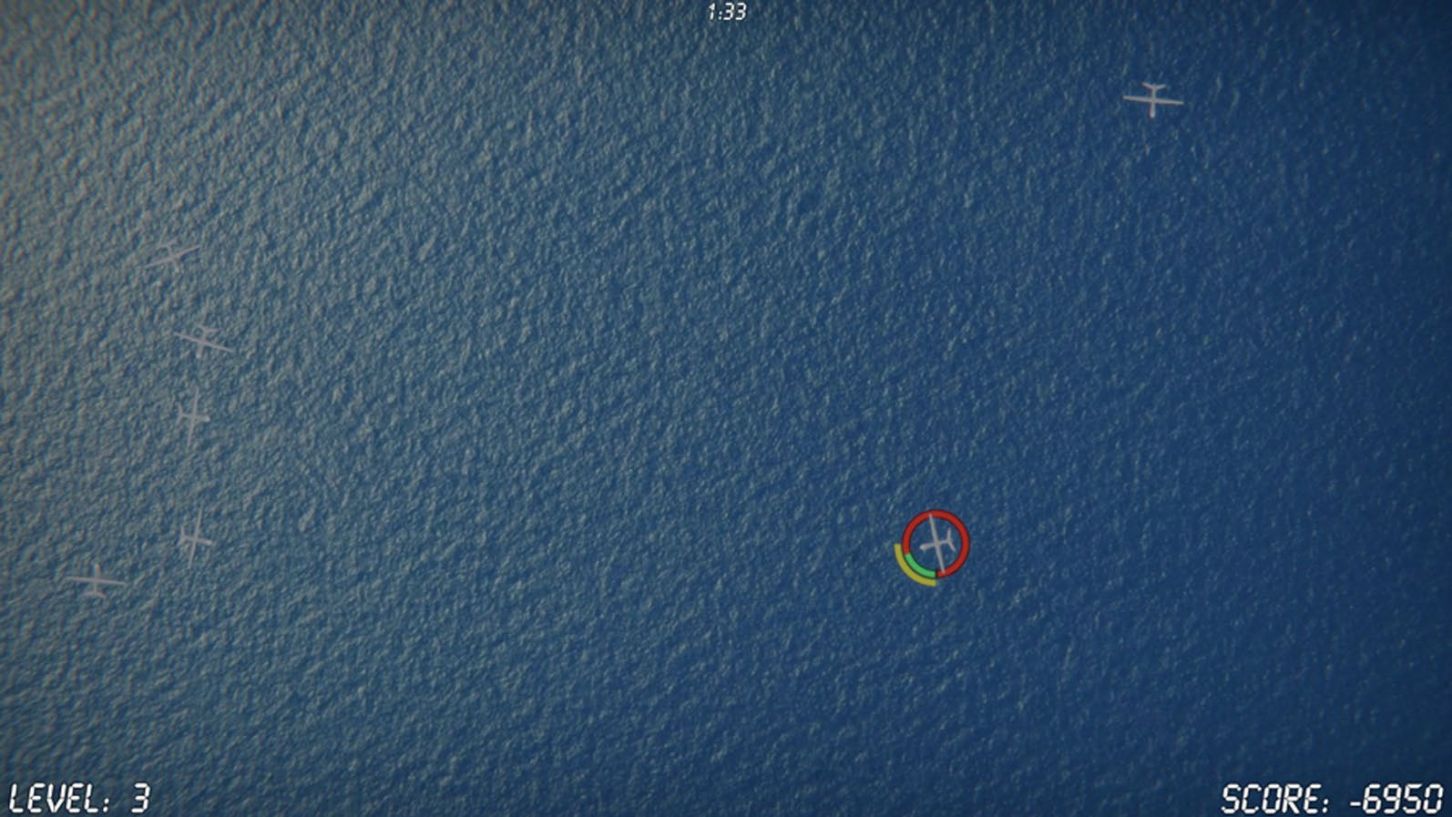
Task display with seven UAVs. Hovering the mouse over a UAV displays the fuel gauge which shows that the fuel level (yellow bar) is not yet in the green region where it can be refueled. The time remaining in the block is displayed at the top, the difficulty at the bottom left, and the score at the bottom right of the screen

The UAV task was developed using the Unity® game engine (Unity Technologies, 2017). The core objective of the task is to refuel small UAV targets that moved around the computer screen at a relatively slow speed of 75 pixels-per-second (i.e., about 1/25-th of the screen width of a standard 1080p monitor at a resolution of 1920 $\times$ 1080 pixels). An illustration of a version of the task is available at https://youtu.be/QcGem6FwnMQ. The task is presented from a birds-eye perspective with a static ocean image shown in the background. UAVs appear as small gray planes seen from above, randomly moving about the full space of the screen (see Fig. 1). Fuel levels depleted at a rate of 5% per second. Clicking on the UAV when fuel entered the lower 25% of the gauge (a “Hit refuel”) refueled the plane and resulted in a score increase. Clicking on a UAV when fuel levels were above 25% (a “False-Alarm Refuel”) resulted in a score deduction. If the fuel fully depleted (a “Miss Refuel”), the UAV crashed, the score was reduced, and a new UAV immediately entered the screen. When a new UAV entered the screen, it began with 70-100% fuel level. Each time a refuel event occurred the refueled plane gained between 50% and 100% of their full tank. When hovered over with the mouse cursor for at least 0.3s a circular fuel-gauge appeared around the UAV depicting the level of remaining fuel in that vehicle and its optimal refueling zone. Participants were allowed one hover-over event to gauge initial fuel levels. Subsequent hover-over events rewarded points (a “Hit Hover Over”) if the remaining fuel level was under 25% or penalized points (a “False-Alarm Hover Over”) if it was over. Each scoring event was briefly presented as a signed points number above the relevant UAV while the total score (shown in the bottom right) was updated. Table 1 shows the points associated with the five different game events. As can be seen, payoffs were maximized by increasing the number of well-timed refuels and decreasing the number of exploding UAVs.

**Table 1:** Payoff scheme

Task event	Points
False-Alarm Hover Over (FAHO)	− 50
False-Alarm Refuel (FAR)	− 500
Hit Hover Over (HHO)	50
Hit Refuel (HR)	2000
Miss Refuel (MR)	− 1000

### Design and procedure

A 3 (task difficulty) x 2 (session) repeated-measures design was used. Task difficulty was varied by adjusting the number of UAV targets that are simultaneously present on the screen. Sessions consisted of 21 blocks. Task difficulty was varied in pseudo-random order across blocks, with each consecutive subset of 3 blocks containing an easy (3 UAV), medium (5 UAV), and hard (7 UAVs) block. Blocks lasted 2 minutes, allowing for a short break of 15 s between blocks, during which the score for different task events was tallied. Participants were given a countdown 5 s to the start of the next block. Three practice blocks were given at the beginning of each session in a fixed easy, medium, hard order, and these were not analyzed. We define a “trial” as the time period between two DRT prompts; hence a trial ranges randomly from 3-5 s according to a uniform distribution.

Upon arrival at the first session of the study, participants were provided with a consent form to sign, acknowledging the purpose of the study and their level of compensation. They were then introduced to the GSR sensors and the DRT task. Instructions were given following the International Standards Organization protocol for DRT measurement of cognitive workload (ISO 17488, 2016). ISO 17488 specifies that participants are instructed to prioritize the primary task (i.e., the UAV task) and the instructions referred to the DRT as the secondary task. For example, participants were instructed to “Please do your best to pay attention to both tasks but recall that your primary task is to monitor the UAVs.” Participants were further read a script that informed them of their goal to refuel each unmanned aerial vehicle (UAV) by hovering to check the fuel level and then left-clicking to refuel, the difficulty levels (easy, medium, hard), and the number of UAVs that pertained to each level (3, 5, and 7, respectively). Participants were also informed that the experiment would consist of 3 practice blocks, during which time they would be allowed to ask questions and receive additional instruction or clarification, followed by 21 test blocks, during which time they would not be allowed to communicate with the experimenter. Finally, they were informed that the blocks would persist for two minutes at a time that the experiment would last approximately 1 h and that they were free to stop for any reason at any time and still receive full compensation of $20 per hour. Following this, participants completed the practice block and the seven experimental sets consisting of an easy, a medium, and a hard block. The second session occurred between one and three days after the first session and consisted only of the practice and experimental blocks. When complete, participants were compensated for their time and thanked for participation.

## Results

### Points and task events

The goal of our first analysis was to establish whether participants were sensitive to the payoff structure of the UAV task. If they are sensitive to payoffs, over the course of the two experimental sessions participants should learn to avoid task events that are associated with a large loss in points and to seek strategies that maximize the points they earn. This analysis was based on the complete data of 46 participants performing 21 experimental blocks on each of two testing days. Note that the number of task events naturally tends to increase with difficulty simply because there are more UAVs on the screen; hence, the effect of difficulty is not of primary interest in this analysis except to the degree that it interacts with day. The same is true of points, beyond the observation that participants were on average able to make use of the greater number of opportunities to earn points as difficulty increased and so the average score increased with difficulty.

**Fig. 2:**
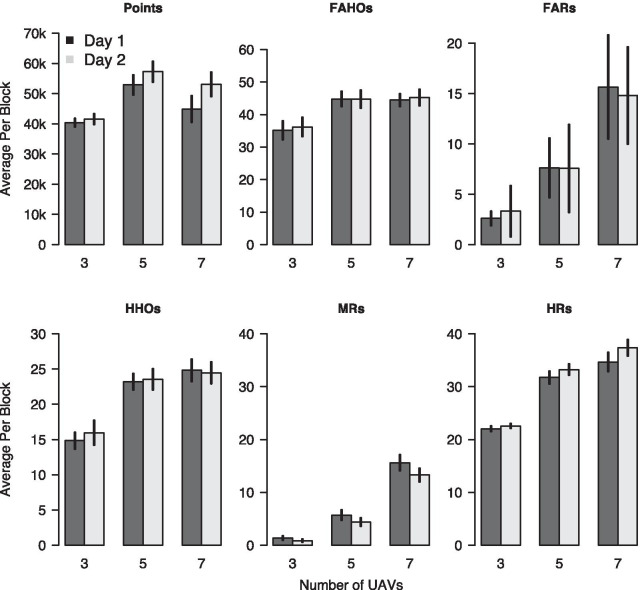
Overview of points earned and frequency of different task events (see Table 1 for definitions) per block for both testing days and the three difficulty conditions. Error bars without caps show mean $\pm 1.96$ SEM

Figure 2 summarizes different aspects of participants’ performance in the UAV task. The top left plot shows the average number of points participants earned in each experimental block on day 1 and day 2 in each of the three difficulty conditions. As can be seen, the number of points earned increased over days in all three difficulty conditions, with average increases being largest in the most difficult condition.

We tested these observations statistically using linear mixed effects models (LMMs; Pinheiro and Bates, 2000). To this end, we created three models with the average number of points earned per block as the dependent variable and a random intercept for each participant. The first model, ${\mathcal {M}}_{diff}$, only included difficulty as a predictor, the second model, ${\mathcal {M}}_{diff+day}$, additionally included an additive effect of day, and the third model, ${\mathcal {M}}_{diff\times day}$, furthermore allowed for an interaction between difficulty and day.

We used the Akaike information criterion (*AIC*; Akaike, 1973) and the Bayesian information criterion (*BIC*; Schwarz, 1978) to compare the different candidate models. The two model selection criteria differ in their assumptions about the true data-generating model; while *BIC* assumes that the true model is among the candidate models, *AIC* does not assume that the true model is included in the set of candidate models. As a consequence, *BIC* penalizes model complexity more heavily and tends to prefer simpler models (see Aho et al., 2014 for a discussion). In most of the model comparisons presented below, both model selection criteria preferred the same model. As the models we consider here are relatively simple, in cases where the two criteria prefer different models, the model preferred by *AIC* might be provided a better approximation to the true data generating model. Both information criteria can be interpreted in terms of the deviation of the *AIC*/*BIC* value of each model in a set of candidate models from the smallest *AIC*/*BIC*. As a rough guideline for the interpretation of *AIC*/*BIC*, differences between 2 and 7 typically indicate that the candidate model is somewhat supported by the data, and differences larger than 7 indicate that the candidate model is not supported by the data (Burnham et al., 2011).

The AIC and BIC values for the different models are presented in Table 2. As can be seen in the first line of the table, *AIC* and *BIC* were smallest for ${\mathcal {M}}_{diff\times day}$, which confirms that the average number of points earned increased from day 1 to day 2, with gains being larger in the most difficult condition.

**Table 2:** Results of model comparison for effect of testing day and difficulty on points and task events (see Table 1 for definitions)

Dependent variable	AIC			BIC		
	${\mathcal {M}}$_diff_	${\mathcal {M}}$_diff+day_	${\mathcal {M}}$_diff×day_	${\mathcal {M}}$_diff_	${\mathcal {M}}$_diff+day_	${\mathcal {M}}$_diff×day_
Average Points	41398	41306	**41272**	41426	41339	**41316**
FAHO	13916	**13913**	13914	**13938**	13941	13953
FAR	12167	12169	**12137**	12189	12197	**12176**
HHO	11924	11923	**11915**	**11946**	11950	11954
MR	8579.6	8446.3	**8431**.**3**	8601.8	8474.2	**8470**.**3**
HR	11076	11038	**11036**	11098	**11066**	11075

Values in bold indicate the best-fitting model

The second plot in the top row of Fig. 2 shows the average number of “False-Alarm Hover Over” (FAHO) events per experimental block on day 1 and day 2 in each of the three difficulty conditions. As can be seen, the number of FAHOs did not change across days. We used generalized linear mixed effects models (GLMs) to assess this observation statistically. Specifically, we fitted the average number of FAHOs using Poisson family models with a logarithmic link function. Similar to the analysis for the average number of points earned, we created three models that allowed for a random intercept for each participant. The results of the formal model comparison are shown in the second row of Table 2. The *AIC* indicated a slight advantage of ${\mathcal {M}}_{diff+day}$ over the other two models, while *BIC* indicated strong support for the simplest model, ${\mathcal {M}}_{diff}$. These formal results align with the observation that the number of FAHOs did not change substantially across days.

The third plot in the top row of Fig. 2 shows the average number of “False-Alarm Refuel” (FAR) events per experimental block across difficulty conditions and days. The plot shows that the number of FARs did not change systematically across difficulty conditions, but rather increased in the easiest condition and decreased in the hardest condition. We again tested these observations using Poisson family GLMs that were specified as for the analysis of the FAHOs. The results of formal model comparison, shown in the third row of Table 2, show a strong preference for ${\mathcal {M}}_{diff\times day}$. However, as ${\mathcal {M}}_{diff + day}$ performed worse than ${\mathcal {M}}_{diff}$ in terms of *AIC* as well as *BIC*, the latter effect is solely driven by the interaction of day and difficulty. Due to the absence of a main effect of difficulty this interaction effect should not be interpreted. These results mean that there was no statistically reliable change in the number of FAR events between testing days.

The first plot in the bottom row of Fig. 2 shows the average number of “Hit Hover Over” (HHO) events per experimental block across difficulty conditions and days. There only appears to be a difference between day 1 and day 2 in the easy condition but not in the remaining difficulty conditions. Our statistical analysis used three GLMs that were specified as the models for the FAHO events. The results of the model comparison are shown in the fourth row of Table 2. *AIC* was equal for ${\mathcal {M}}_{diff}$ and ${\mathcal {M}}_{diff + day}$. That is, there was no evidence for a main effect of day. *BIC*, on the other hand, was smaller for ${\mathcal {M}}_{diff}$ than for the two alternative models. These results confirm that there was no change in the number of HHO events between testing days.

The second plot in the bottom row of Fig. 2 shows the average number of “Miss Refuel” (MR) events per experimental block across difficulty conditions and days. The average number of MRs decreased from day 1 to day 2 in all difficulty conditions, with decreases being largest in the most difficult condition. We again tested these observations statistically by comparing three GLMs. This model comparison confirmed the qualitative observations; both *AIC* and *BIC* were substantially smaller for ${\mathcal {M}}_{diff \times day}$ compared to the other models.

Finally, the third plot in the bottom row of Fig. 2 shows the average number of “Hit Refuel” (HR) events per experimental block across difficulty conditions and days. The average number of HRs increases from day 1 to day 2, with gains being largest in the most difficult condition. A formal model comparison confirmed these observations. *AIC* indicated a strong advantage of ${\mathcal {M}}_{diff + day}$ over ${\mathcal {M}}_{diff}$ but only a negligible advantage of ${\mathcal {M}}_{diff \times day}$ over ${\mathcal {M}}_{diff + day}$. *BIC* indicated a strong advantage of ${\mathcal {M}}_{diff + day}$ over both competitor models.

Taken together, the analysis of the task data indicates that participants learned to improve the number of points earned. This increase from day 1 to day 2 was principally driven by those UAV-task events that were associated with considerable penalties and rewards, indicating that participants adjusted their behavior according to the payoff manipulation. Subsidiary analyses of arcsine-transformed mean number of events per participant using Bayesian ANOVAs confirmed these conclusions (see Supplementary Materials).

### Detection-response task

We carried out our analysis of the DRT data in two steps. In a first step, we evaluated whether DRT behavior was sensitive to the workload manipulation in the UAV task. In a second step, we explored the relationship between UAV-task events and DRT behavior. We were interested in how different types of task events affect RTs and omissions on the DRT, and how these relationships evolve as participants gain more experience with the UAV task.

#### Difficulty manipulation

**Fig. 3:**
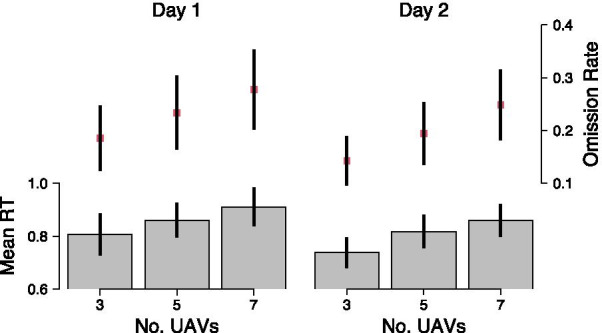
Mean response time (RT) and omission rate for the three task difficulty levels, separately for the two testing days. Error bars show mean $\pm 1.96$ SEM

Figure 3 shows the effect of the number of UAVs on mean RTs and omission rates. As can be seen, both mean RTs and omission rates increased as the number of UAVs increased, which suggests that the increased difficulty in the UAV task reduced participants’ DRT performance. Moreover, RTs and omission rates decreased from day 1 to day 2 in all difficulty conditions, suggesting that cognitive workload decreased as participants became more skilled at the task. RTs, for instance, decreased on average by 5.4% in easy blocks and by 3.7% in medium and high difficulty blocks. We tested these qualitative observations statistically using LMMs for the log-RTs and binomial family GLMs for the omission rates. We specified the same three models of each type as in earlier analyses that included random intercepts for participants and used log-RTs or omission rates as dependent variable. The results of the model comparison are shown in Table 3. For log-RT (top row), both *AIC* and *BIC* indicated ${\mathcal {M}}_{diff+day}$ as the best model, which supports the observation that log-RT decreased across days and was longer for more difficult conditions but the change in log-RT between days was comparable for all difficulty conditions in a proportional sense (as additivity on a log scale indicates a constant proportional difference).

**Table 3:** Results of model comparison for effect of testing day and difficulty on DRT performance

Dependent variable	AIC			BIC		
	${\mathcal {M}}$_diff_	${\mathcal {M}}$_diff+day_	${\mathcal {M}}$_diff×day_	${\mathcal {M}}$_diff_	${\mathcal {M}}$_diff+day_	${\mathcal {M}}$_diff×day_
Log-RT	61493	**61399**	61403	61536	**61451**	61472
Omissions	43725	43577	**43569**	43761	**43621**	43632

Values in bold indicate the best-fitting model

For omissions (bottom row in Table 3), *AIC* preferred ${\mathcal {M}}_{diff + day}$ over ${\mathcal {M}}_{diff}$ and ${\mathcal {M}}_{diff\times day}$ over ${\mathcal {M}}_{diff + day}$, which suggests that omission rate increased with increasing task difficulty and decreased across days, but the decrease was differently affected by different difficulty conditions. *BIC*, on the other hand, suggests ${\mathcal {M}}_{diff + day}$ as the best fitting model. Nevertheless, both model selection criteria agree that task difficulty and testing day both affected omission rates.

Taken together, these results confirm that participants’ DRT performance was sensitive to the difficulty manipulation, with higher workload in the more difficult condition resulting in decreased DRT performance. Moreover, there appear to be clear practice effects reflected in improved DRT performance on day 2 compared to day 1.

### Predicting workload fluctuations

In the second step of our analysis, we investigated how the UAV task affects cognitive workload on a real-time basis, as reflected in fluctuations in DRT performance. In particular, we were interested in predicting cognitive workload based on recent events in the UAV task. As advocated by several authors in recent years (Hastie et al., 2009; Koul et al., 2018; Yarkoni and Westfall, 2017), we took a cross-validation approach to our statistical analysis to safeguard generalizability of our results. We first identified a number of observable task events that might potentially affect workload as measured by the DRT. Subsequently, we refined and complemented these predictors with other variables that should reflect psychological influences on participants’ cognitive workload. To guarantee that sufficient data were available for all these analyses, we removed the data of all participants who failed to respond to more than 45% of DRT prompts in two or more difficulty conditions. This resulted in the removal of seven participants on day 1 and five participants on day 2.

#### Time on task

**Fig. 4:**
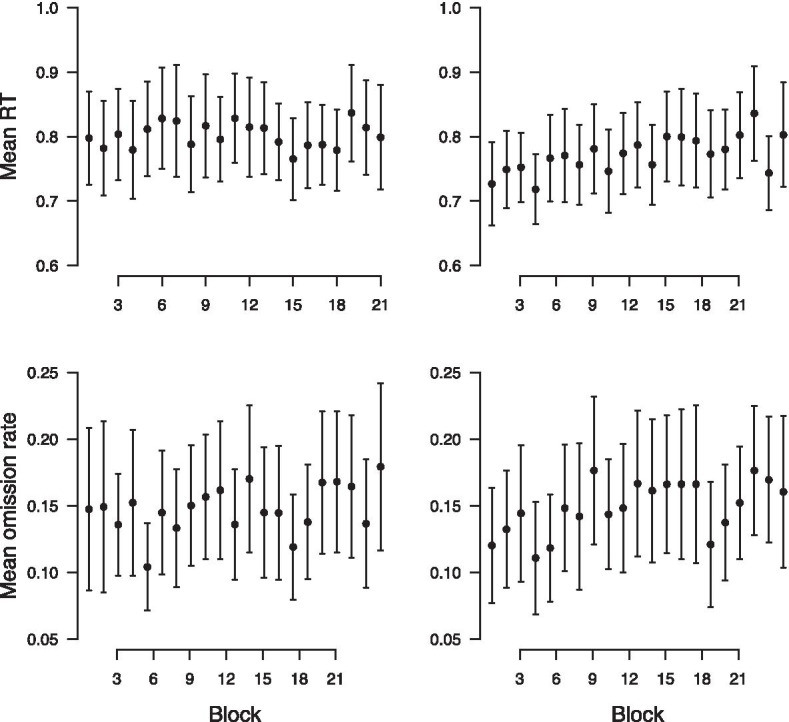
Development of DRT performance across blocks. Top row: mean RT across blocks. Bottom row: mean omission rate across blocks. Error bars show mean $\pm 1.96$ SEM

Before performing the predictive analysis, we investigated time on task, as we expected that fatigue might affect DRT performance independent of specific UAV task events and cognitive workload. Figure 4 shows the development of DRT performance across blocks. The top row shows how mean RT changes across experimental blocks on day 1 (left) and day 2 (right). There does not appear to be a systematic relationship between experimental block and RT on day 1, but on day 2 mean RT appears to increase across blocks, consistent with a fatigue effect. The apparent lack of a relationship between RT and block on day 1 might be due to any fatigue effect being masked by learning in the UAV task that decreased its cognitive demands.

The bottom row shows how mean omission rate changes across experimental blocks. There appears to be a slight increase in omission rate across blocks on both days. However, this tendency is difficult to interpret due to the high standard errors in the observed mean omission rates.

To assess these observations statistically, we used LMMs models to test the relationship between RT and block, and binomial family GLMs to test the relationship between omission rate and block. In light of the results for the relationship between difficulty and RT/ omission rate above, we considered three different models with RT/ omission rate as dependent variables and random intercepts for each participant. The first model ${\mathcal {M}}_{diff + day}$ only used day and difficulty as predictors. The second model, ${\mathcal {M}}_{diff + day + block}$, additionally allowed for an effect of block. The third model ${\mathcal {M}}_{diff + day \times block}$, furthermore allowed for an interaction effect between block and day. The results of the model comparisons are shown in Table 4. For the models with RT as dependent variable, shown in the top row, *AIC* indicated ${\mathcal {M}}_{diff+day \times block}$ as the best model, supporting an effect of block on RT, but only on day 2. *BIC*, on the other hand, preferred ${\mathcal {M}}_{diff+day + block}$, which suggests that block affected RT equally on both days. The results for the models with omissions as dependent variable are shown in the bottom row of Table 4; both *AIC* and *BIC* indicate that ${\mathcal {M}}_{diff + day + block}$ accounts best for the observed data, which means that omission rates increased equally across blocks on both days.

**Table 4:** Results of model comparison for effect of block on DRT performance

Dependent variable	AIC			BIC		
	${\mathcal {M}}_{diff+day}$	${\mathcal {M}}_{diff+day + block}$	${\mathcal {M}}_{diff + day \times block}$	${\mathcal {M}}_{diff}$	${\mathcal {M}}_{diff+day}$	${\mathcal {M}}_{diff\times day}$
Log-RT	57652	57641	**57620**	57704	**57701**	57689
Omissions	34573	**34541**	34541	34617	**34594**	34602

Taken together, these results suggest the presence of fatigue effects that led to a decline in DRT performance across blocks. The effect of block on RT might have been mitigated by learning effects on day 1, leading to an interaction between block and testing day.

#### Prediction from UAV-task events occurring at different timescales

The goal of our first cross-validation analysis was to identify which observable UAV-task events significantly affected subsequent cognitive workload. The outcomes of this analysis were then used to generate refined predictors that not only used observable task events but also included proxies of participants’ dynamic internal representation of the UAV task. For this initial analysis, we considered the UAV-task events FAHO, HHO, FAR, MR, and HR. These predictors were defined as in our previous analysis and were based on the occurrence or non-occurrence of the relevant task event in a predefined time period.

There is no a priori way of determining the timescale on which task events affect workload. That is, we do not know whether the occurrence of a task event will increase cognitive workload and hence affect DRT performance, 3, 4, or even 5 s later. Therefore, we repeated our analysis three times, once for RT and omissions if an event of a specific type occurred up to 3 s prior to the DRT prompt, once for the case that an event occurred up to 4 s prior to the DRT prompt, and once for the case that an event occurred up to 5 s prior to the DRT prompt. We focused on the 3-5-s intervals because they spanned the range of intervals between DRT stimuli.

**Fig. 5:**
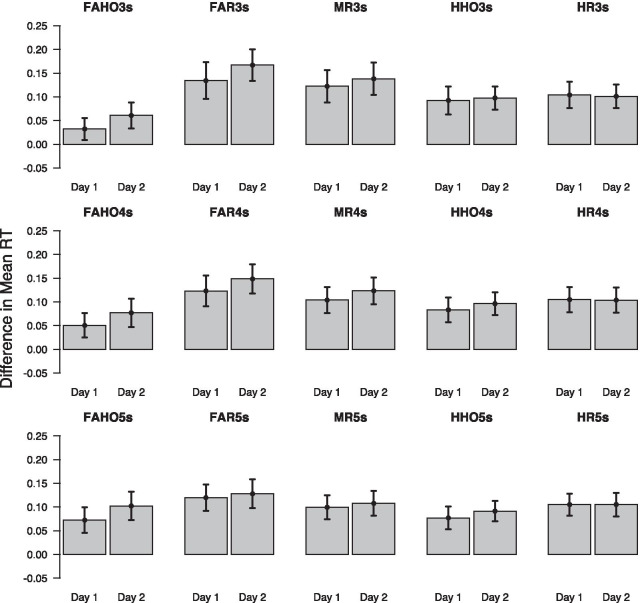
Effect of different UAV-task events on response time (RT) for different time windows prior to the DRT prompt. Top row: 3-s time window; middle row: 4-s time window; bottom row: 5-s time window. Error bars show mean difference ± 1.96 SEM

Figure 5 shows how different UAV events affect RT on different timescales. Each plot shows the difference in mean RT between trials on which the specific event occurred and trials on which the event did not occur, for day 1 and day 2. The top row shows the results for events occurring up to 3 s prior to the DRT prompt, the middle row shows the results for a 4-s time window, and the bottom row shows the results for a 5-s time window. As can be seen, on both days the occurrence of any event during any of the three time windows appears to delay the detection response. Differences between different classes of events appear to be relatively small and differences between day 1 and day 2 for the same type of event seem negligible. However, it should be noted that different types of events might be considerably correlated, either because some types of game events logically precede others, or because several different events occur within the same time window. We will, therefore, rely on multivariate statistical analyses to disentangle these correlations.

**Fig. 6:**
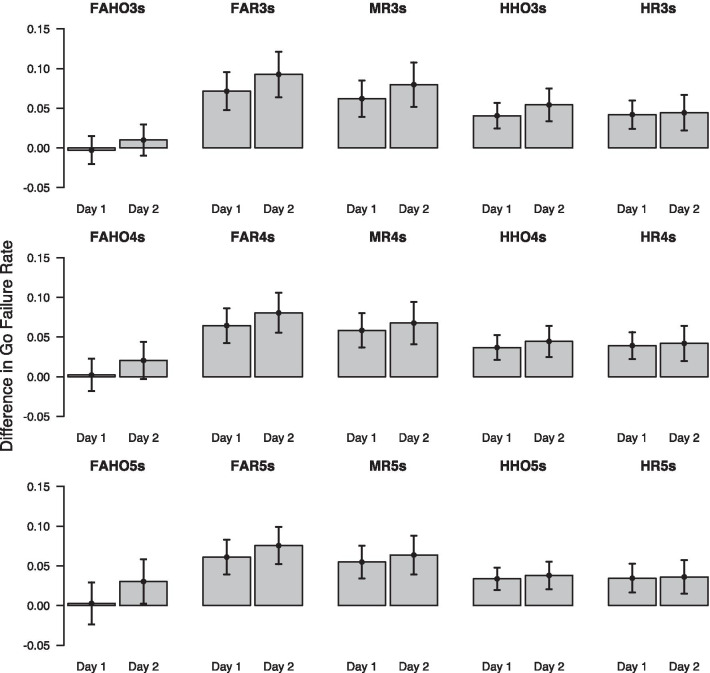
Effect of different UAV events on omissions for different time windows prior to the DRT prompt. Top row: 3-s time window; middle row: 4-s time window; bottom row: 5-s time window. Error bars show mean difference ± 1.96 SEM

Figure 6 shows how different UAV events affect omission rates on different timescales. Each plot shows the difference in mean omission rate between trials on which the specific event occurred and trials on which the event did not occur, for day 1 and day 2. Similar to the results for the effect on RT, all event types except for FAHO and HHO seem to affect omissions to the same extent on both days, and differences between these event types seem negligible. FAHO and HHO, on the other hand, appear to influence omissions more on day 2 than on day 1. However, we again note that the occurrence of different event types is highly correlated and a multivariate analysis approach is needed to disentangle these correlations.

As we found clear learning effects over days, which may change what events, if any, are predictive, we analyzed the data for each testing day separately. We split the available data of all participants for each day into five non-overlapping subsets of participants, the cross-validation folds. The data were split into folds with approximately equal numbers of participants. Each fold served in turn as the training set and the remaining four subsets served as cross-validation sets.

The two outcome variables of the DRT, RTs, and omissions are known to be intricately linked (e.g., Wickelgren, 1977; Ratcliff, 1978) and statistical models should ideally account for both quantities simultaneously. One approach to simultaneous modeling is offered by two-part models (TPMs) for semi-continuous data (Aitchison, 1955; Farewell et al., 2017). For our DRT data, such a model would assume a log-normal distribution for the RT data and represent omissions as a point-mass at zero. Unfortunately, easy-to-use software implementations for such log-normal TPMs have only become available recently and lack the capabilities required for our cross-validation analyses. The GLMMadaptive R-package (Rizopoulos, 2020), which we will use here, implements maximum-likelihood fitting routines for log-normal TPMs. However, predictions for new cases can only be generated for the log-normal intensity variable (i.e., log-RTs) but not for the occurrence variable (i.e., omissions), which means that our cross-validation analysis is limited to the RT data. We therefore also consider a second analysis approach that applies cross-validation separately to LMMs for the log-RTs and to binomial-family GLMs for the omissions.

In our first set of analyses, we consider five types of UAV-task events as predictors (FAHO, HHO, FAR, MR, and HR) that might influence cognitive workload and thus affect log-RT and omissions. We considered experimental block as a potential proxy for learning and fatigue effects that might influence log-RT. Moreover, based on the results of our previous analyses, we also included difficulty as a potential predictor. These seven potential predictors can be combined into $2^8=128$ different sets of predictors. This would yield a total of $128^2 = 16384$ models that implement only the possible additive (i.e., no interactions) combinations of fixed effects and random intercepts. Further computational issues arise from the fact that most of our predictors are discrete with only a small numbers of observations in some cells, which means that estimates of interaction effects are notoriously numerically unstable. Hence, to keep the computational costs at a manageable level and guarantee numerical stability, we only used models with additive fixed effects and random subject intercepts. Moreover, we only considered all possible combinations of the five UAV-task events as predictors, with difficulty and experimental block always included as predictors. In addition, we considered four baseline models that included only block and difficulty, only block, only difficulty, or only an overall intercept as predictors.

We fit each model to the training set consisting of four of the cross-validation folds and used the resulting parameter estimates to predict the data of the remaining fold. To measure predictive performance, we computed the mean squared deviation (MSD) between observed and predicted log-RT or omission rate for each cross-validation fold, and normalized it by subtracting the average MSD across all five folds (NMSD). Lower values of NMSD indicate better predictive performance. This analysis was repeated for time windows of 3 s, 4 s, and 5 s prior to the DRT prompt.

**Fig. 7:**
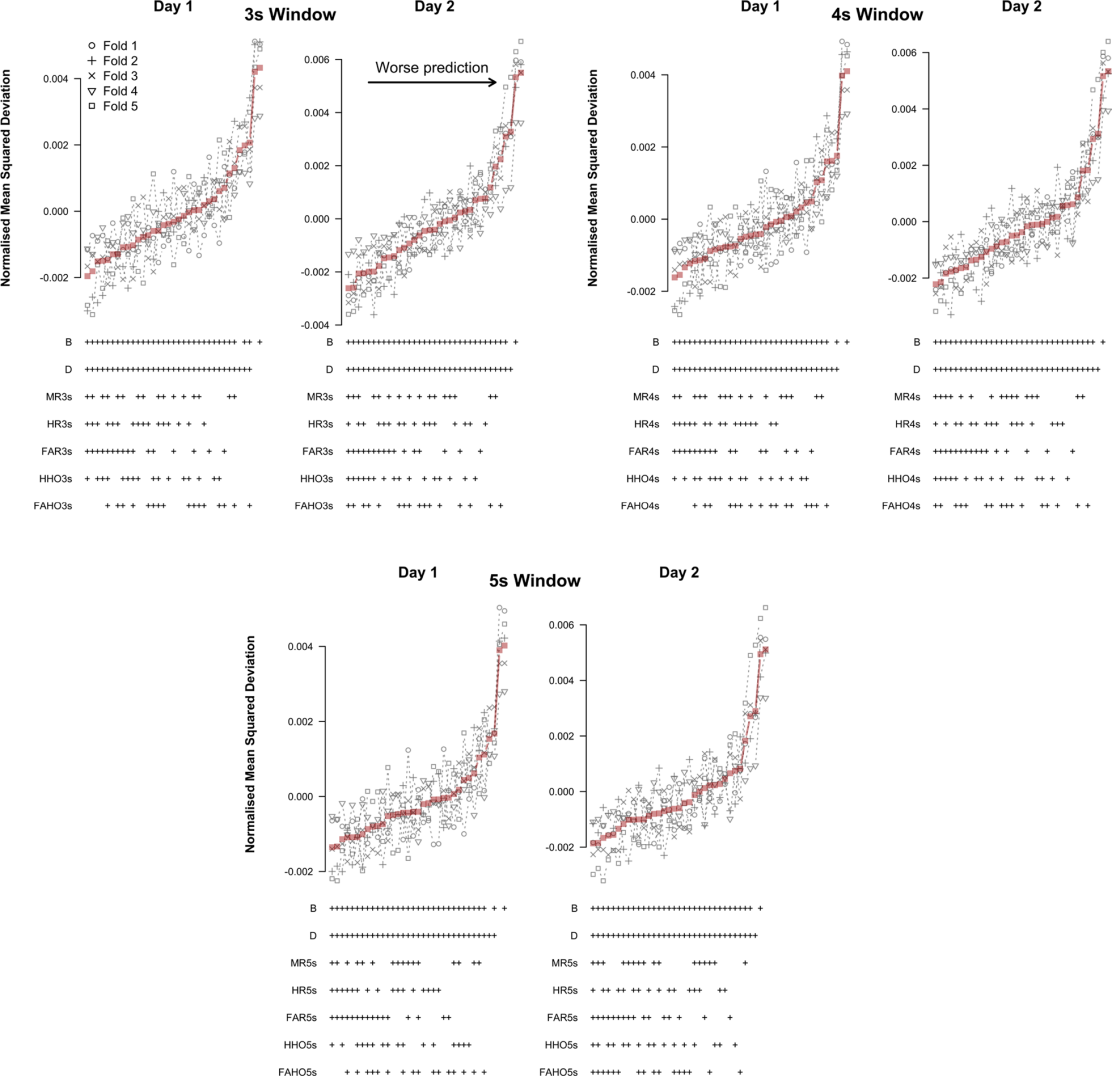
Cross-validation results based on TPMs for different UAV-task event predictors of log-RT. Normalized mean squared deviations for different LMMs for different time windows prior to the DRT prompt are shown. Top row: 3-s time window; middle row: 4-s time window; bottom row: 5-s time window. Left column: results for day 1; right column: results for day 2. Red squares show the mean across folds

Figure 7 shows the results of the cross-validation analysis based on TPMs for different time windows and testing days. Results are displayed in order of increasing mean NMSD across folds. A ‘+’ in the label below each data point indicates that the corresponding variable was included as a predictor in the model. NMSD showed similar patterns on both testing days and across time windows, which suggests that UAV-task events can affect DRT performance over a time span of several seconds. NMSD was considerably lower for models that included difficulty and experimental block as predictors than for the baseline models that did not include these predictors. This means that both predictors convey essential predictive information. Among the five UAV events, only FAR appears consistently in all models with good predictive performance, for all time windows and testing days. FAR should therefore be considered an essential predictor in the context of TPMs. For time windows of 3s and 4s, HHO is also included in a large number of models with low NMSD on day 2 and FAHO consistently appears in the best models models on day 2 for the 5s window. Overall, these patterns were consistent across cross-validation folds.

**Fig. 8:**
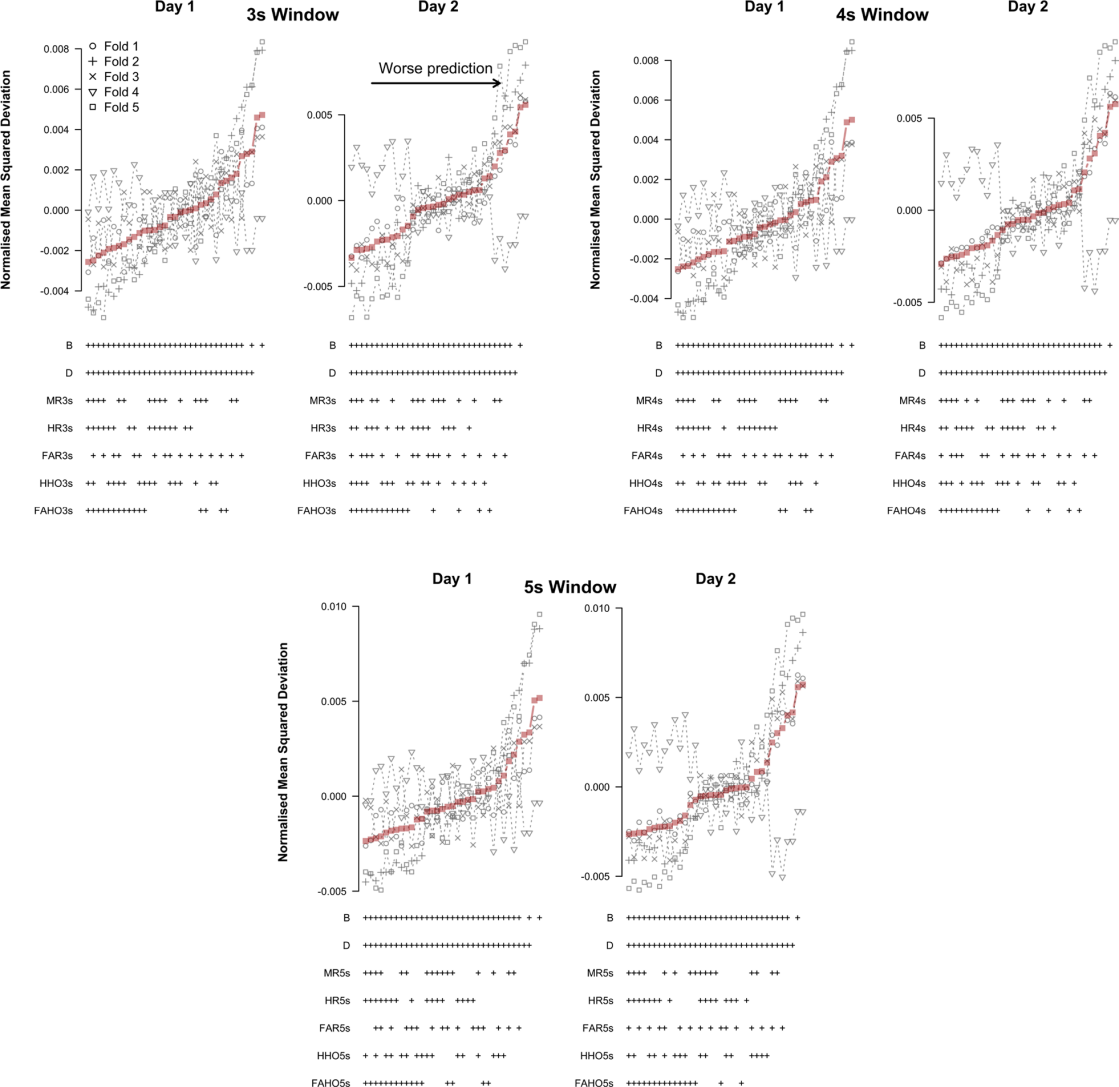
Cross-validation results based on LMMs for different UAV-task event predictors of log-RT. Normalized mean squared deviations for different LMMs for different time windows prior to the DRT prompt are shown. Top row: 3-s time window; middle row: 4-s time window; bottom row: 5-s time window. Left column: results for day 1; right column: results for day 2. Red squares show the mean across folds

Figure 8 shows the results of the cross-validation analysis based on LMMs for different time windows and testing days. Similar to the TPMs, NMSD showed comparable patterns on both testing days and across time windows. Moreover, NMSD was again considerably lower for models that included difficulty and experimental block as predictors than for the baseline models that did not include these predictors. This confirms that both predictors convey essential predictive information. Among the five UAV events, only FAHO appears consistently in all models with good predictive performance, for all time windows and testing days. HR is also included in a sizeable number of models with low NMSD on day 1, but also appears in some models with high NMSD, particularly on day 2. We also note that fold 4 produced results that were inconsistent with the other folds for the analysis of day 2 (Fig. 8).

**Fig. 9:**
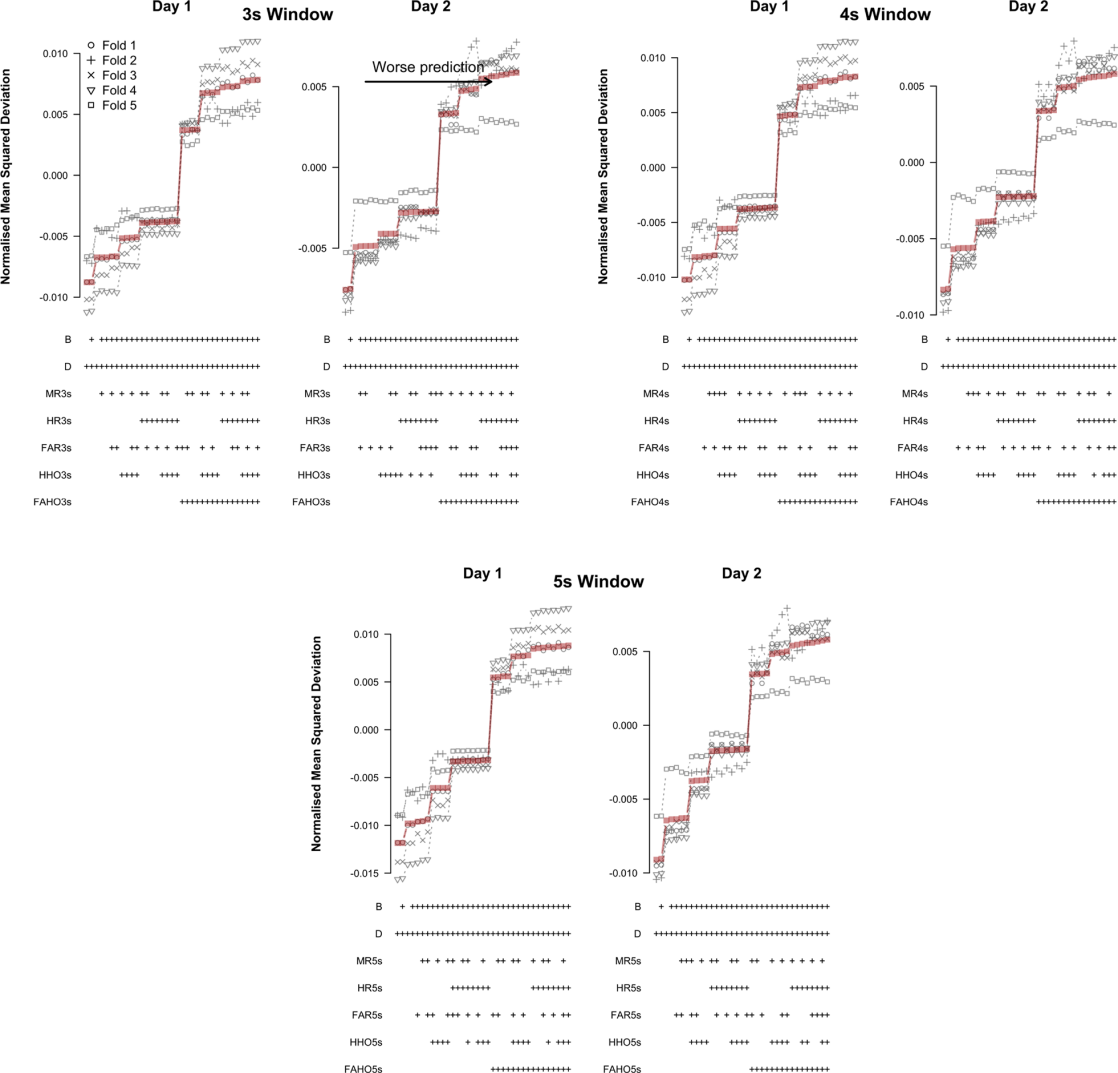
Cross-validation results based on binomial-family GLMs for different UAV-task event predictors of omissions. Normalized mean squared deviations for different binomial family generalized linear mixed effect models for different time windows prior to the DRT prompt are shown. Top row: 3-s time window; middle row: 4-s time window; bottom row: 5-s time window. Left column: results for day 1; right column: results for day 2. Red squares show the mean across folds

Figure 9 shows the results of the cross-validation analysis based on binomial-family GLMs for different time windows and testing days. Predictive performance was measured in terms of NMSD between the observed and predicted probability of an omission. As for the TPMs and LMMs, the NMSD showed similar patterns on both testing days and across time windows. However, in contrast to the TPMs and LMMs, none of the task events were consistently included in the models with low NMSD. In fact, task events that were good predictors for log-RTs, FAHO, HHO, and FAR performed poorly for omissions. The models with the lowest NMSD only included difficulty and experimental block as predictors.

Taken together, the results of our first set of cross-validation analyses suggest that participants’ DRT performance is strongly influenced by global levels of difficulty and fatigue. Of the UAV events considered, only FAHO, HHO, and FAR seems to have a moderate influence on RTs in the DRT. A possible interpretation of this effect is that, as their workload increases, participants tend to lose track of the state of the different UAVs. To compensate for this loss of situational awareness, they need to hover over the UAVs to update and refresh their mental representation of the situation. In the next section, we investigate this suggestion by investigating the predictive ability of statistics constructed to measure participants’ situational awareness with respect to fuel load.

#### Refined predictors

We considered six new predictor variables in our second cross-validation analysis, in addition to difficulty and block. As the results of the previous cross-validation analysis were similar for different time windows, and were slightly more stable across folds for shorter time windows, we considered only events within a 3-s time window.

Our initial cross-validation analysis suggested that requesting fuel information is indicative of increased cognitive workload. More fine-grained information about a participant’s cognitive workload might be provided by the frequency with which that person requests fuel information. Participants might attempt to compensate for a loss of situational awareness by requesting fuel information more frequently, which should result in an increased number of HHO and FAHO events within a certain time period. Therefore, we considered the number of HHO events within the last 3 s, “#HHO3s,” and the number of FAHO events within the last 3 s, “#FAHO3s” as new predictors. Our initial cross-validation analysis further suggested that falsely attempting to refuel UAVs that have not yet reached a critical fuel level. The frequency of such FAR events within a given time window might again provide more fine-grained information about cognitive workload. We therefore included the number of FAR events within the last 3 s, “#FAR3s,” as another predictor.

The fourth and fifth potential predictors were also related to participants’ knowledge of fuel levels, but focused on the fact that as participants received a particularly high penalty for exploding UAVs, so their workload should mostly be driven by the UAVs with the lowest fuel levels. If they maintain an accurate mental representation of the current situation, participants should mostly request fuel information for the UAVs with the lowest fuel levels. Hence, our fourth potential predictor was the ordinal position of the UAV participants hovered over most recently, “OrdFuel.” That is, did the last UAV checked in the 3 s before a DRT response have the lowest fuel (OrdFuel = 1), the second lowest (OrdFuel = 2), and so on.

Multiple UAVs reaching a critical fuel level in a short period of time should increase participants’ cognitive workload considerably more than a single UAV reaching a critical fuel level, as that might leave insufficient time to refuel all of them before one or more crashed. Our fifth new predictor combines the fuel level of all UAVs on screen, but gives more weight to lower fuel levels, “WeightFuel.” This computation was based on the Minkowski distance of the vector of fuel levels from 0, normalized by the number of UAVs:

1 $$\begin{aligned} {\text {WeightFuel}} = \left( \sum _{k=1}^{K} \frac{f_k^{p}}{K}\right) ^{1/p}, \end{aligned}$$

where *K* is the total number of UAVs on screen and $f_k$ is the fuel level of the *k*th UAV. Here we set $p=1/4$ (we discuss this choice further below).

We added one final, and somewhat different, potential predictor, whether there was a omission on the previous DRT trial, “MissPrevDRT,” in response to the overall poorer prediction of DRT omissions in the previous cross-validation analysis. An omission on the previous trial might predict an omission or a slower RT on the present trial, due to a period of sustained higher workload. However, an omission on the present trial might also be due to reasons not directly related to workload, such as the occurrence of periods where participants neglected instructions to respond to the DRT and instead responded only to the UAV task. To the degree that the latter process was in play, we might expect MissPrevDRT to predict omissions but not RT.

As information about the preceding trial was not available for the first trial of each block, we excluded these trials from all further analysis steps. Moreover, the data recording system used for the experiment only logged information about UAVs when participants interacted with a UAV. This means that we could only compute the new predictor variables for trials on which participants had interacted with a UAV within a period before being presented the DRT prompt. We therefore removed all trials on which participants had not interacted with a UAV or on which the last interaction with a UAV occurred more than 100s before the DRT prompt. This resulted in the removal of only a small number of trials, a total of 280 from the dataset for day 1 and a total of 253 from the dataset for day 2.

We used the same cross-validation setup as in the previous analysis to test whether any of the five predictors conveys additional information about participants’ DRT performance. We again considered all possible combinations of the six predictors together with difficulty and experimental block. In addition, we considered four baseline models that included only difficulty and experimental block as predictors, only difficulty, only experimental block, or only an overall intercept.

**Fig. 10:**
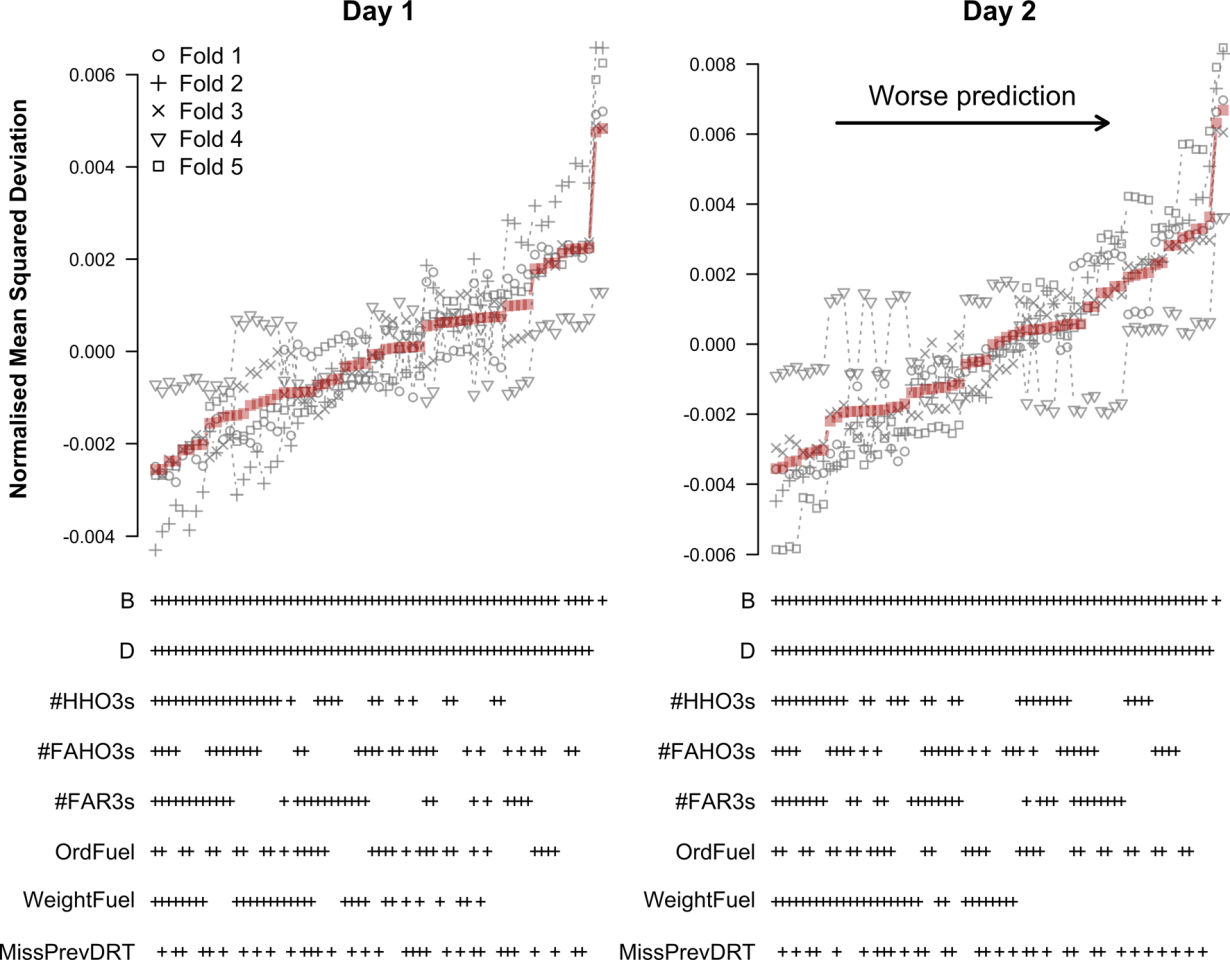
Cross-validation results for predicting log-RT with a log-normal TPM based on refined predictors

Figure 10 shows the results for the TPMs. Models are ordered by increasing average NMSD. As can be seen, models that included block and difficulty in addition to the refined predictors performed better than the baseline models that did not include any refined predictors. Among the refined predictors, #HHO3s, #FAR3s, and WeightFuel were consistently included in the models with the lowest average NMSD on both testing days. These results hold across all cross-validation folds on day 1. On day 2, however, this pattern only holds for four of the five folds. In fold 4, the best models consistently included OrdFuel and #FAR3s as predictors.

**Fig. 11:**
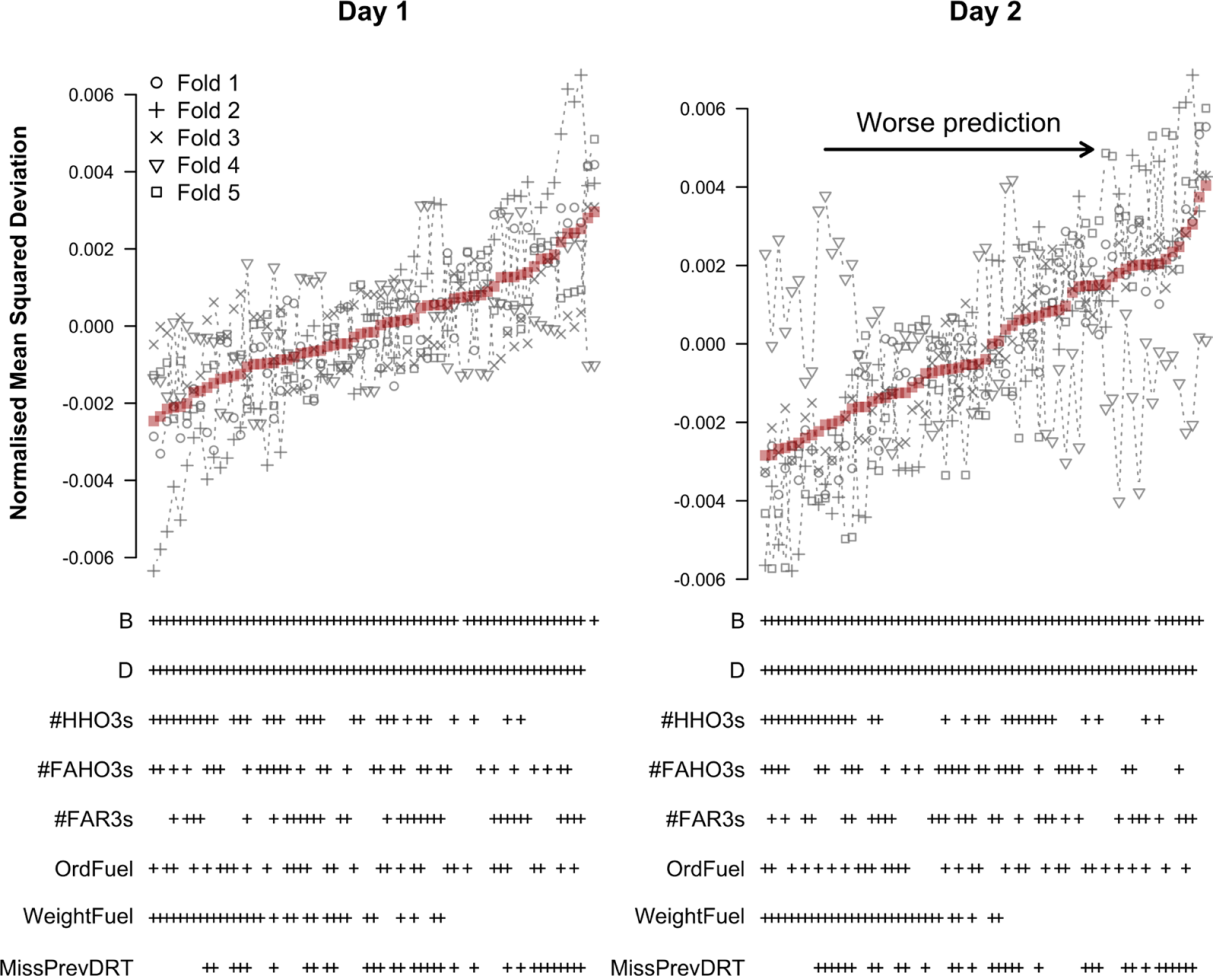
Cross-validation results for predicting log-RT with a LMM based on refined predictors

Figure 11 shows the LMM results for predicting log-RT. The results match those for the TPMs closely. Among the refined predictors, #HHO3s and WeightFuel were consistently included in the models with the lowest average NMSD on both testing days. These results again hold across all cross-validation folds on day 1. However, on day 2 fold 4 again showed a deviating pattern; the best models only consistently included #FAR3s. Nevertheless, by and large, #HHO3s and WeightFuel were most consistently included in the models with the lowest NMSD across folds.

**Fig. 12:**
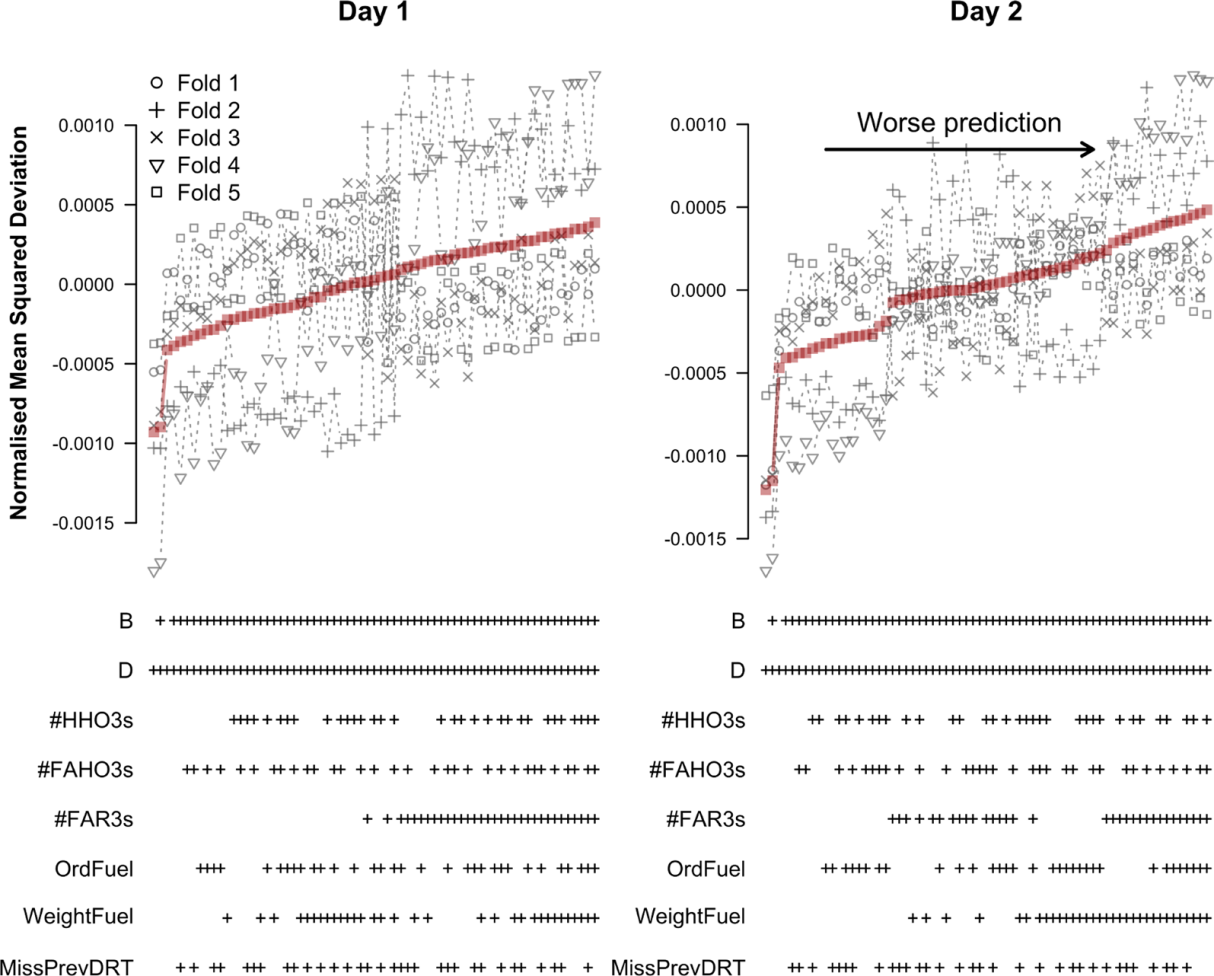
Cross-validation results for predicting omissions based on refined predictors

The results for predicting omissions are shown in Fig. 12. Similar to the results of our initial analysis, the best models for predicting omissions only included block and difficulty as predictors. Additionally including any of the refined predictors led to a steep increase in NMSD. These patterns were consistent across testing days and folds.

**Fig. 13:**
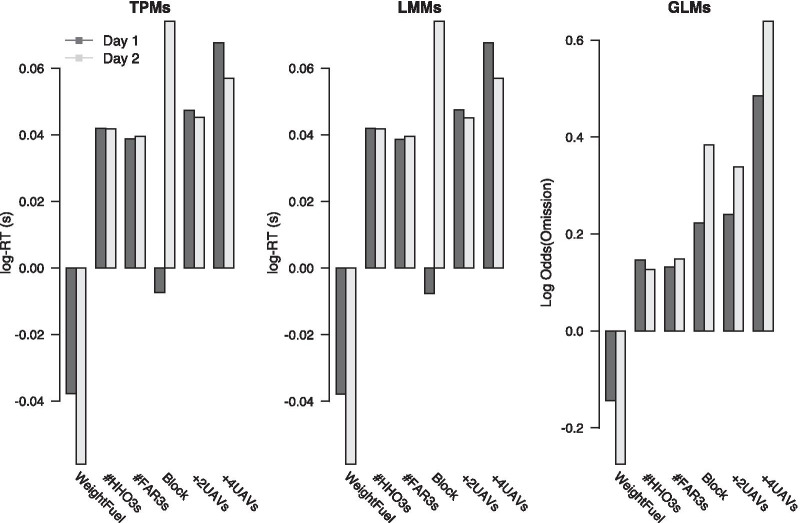
Approximate effect size of different predictors on log-RT and log odds of omissions. Bars show the unstandardized beta weights from a log-normal TPM for response time (log-RT), from a LMM for response time (log-RT) and a GLM for log odds of omissions. Predictors WeightFuel, #HHO3s and #FAR3s were z-standardized within each participant

Figure 13 shows approximate effect sizes for the three refined predictors that performed best for log-RT and/or omissions, and for block and difficulty. The left panel shows the unstandardized beta weights from a log-normal TPM with RT as dependent variable and WeightFuel, #HHO3s, #FAR3s, block, and difficulty as predictors, and a random intercept for each participant. WeightFuel, #HHO3s, and #FAR3s were z-standardized within each participant so that beta weights indicate the change in predicted log-RT if the predictor value increases by 1 SD. Difficulty was entered as a factor with the case of 3 UAVs as the reference level, which is absorbed in the intercept term of the model, and levels $+2$UAVs and $+4$UAVs representing the cases of a total of 5 and 7 UAVs, respectively. The bar for block shows the total effect size for completing 21 blocks. Black bars show the beta weights for day 1, and gray bars show the beta weights for day 2.

As can be seen, effects were similar in size on both testing days for all predictors except for WeightFuel and difficulty. WeightFuel had a negative effect on RT, with a one standard deviation increase in weighted fuel levels decreasing log-RT by about 40ms on day 1 and by about 60ms on day 2. #HHO3s and #FAR3s had a slightly positive effect, with each additional HHO and FAR event increasing log-RT by about 40ms. Block had a negligible effect on RT on day 1 and a considerable positive effect on RT on day 2, resulting in an increase in log-RT by about 75ms after 21 blocks. Increasing the number of on screen UAVs by 2 to a total of 5 increased RT by about 45ms, and increasing the number of on-screen UAVs by 4 to a total of 7 increased RT by 67ms on day 1 and 57ms on day 2.

The middle panel of Fig. 13 shows the unstandardized beta weights from a LMM with log-RT as the dependent variable and the same predictors as in the TPM. The patterns and effect sizes for all predictors matched those for the TPM to four decimal places.

The right panel of Fig. 13 shows the unstandardized beta weights from an binomial family GLM with log odds of omission as the dependent variable.^1^ Predictors were specified as for the LMM. Results were similar to those for log-RT. WeightFuel had a sizeable negative effect on the log-odds of an omission that was more pronounced on day 2 than on day 1. Experimental block, $+2$UAVs, and $+4$UAVs considerably increased the log odds of an omission, and these effects were more pronounced on day 2. Finally, increases in the number of HHO and FAR events had a comparatively smaller effect on the log odds of an omission.

We used TPMs to directly compare the predictive quality of our refined predictors to the event-based predictors from our initial analysis. Within the framework of our experimental task, a direct manifestation of cognitive overload is the explosion of a UAV. Participants are most heavily penalized for failing to refuel a UAV in time and should therefore prioritize refueling above all other game activities. Hence, the number of exploded UAVs is an observable indicator of cognitive overload. We used TPMs to assess whether our refined predictors were better able to predict cognitive overload than the simple event-based predictors used in our initial analysis. To this end, we formally compared Poisson-family zero-inflation models that used either the best event-based predictors or the best refined predictors to predict the number of exploded UAVs within a 5-s time window *after* the DRT prompt had been presented. The first model included the event-based predictors that indicated whether at least one HHO, FAHO, FAR, or HR event had occurred 3s prior to the DRT prompt. The model additional included experimental block and difficulty as predictors to control fatigue effects and the effect of task difficulty. The second model included WeightFuel and the number of HHO and FAR events within a 3-s time window prior to the DRT prompt. The model again included experimental block and difficulty as predictors to control fatigue effects and the effect of task difficulty. Both models were compared separately for each testing day. For day 1, the formal model comparison yielded an AIC value of 32899 and a BIC value of 32934 for the simple event-based predictors, and an AIC value of 24547 and a BIC value of 24578 for the refined predictors. That is, the refined predictors were considerably better at predicting cognitive overload up to 5s after the DRT prompt. For day 2, the formal model comparison yielded an AIC value of 29025 and a BIC value of 29060 for the simple event-based predictors, and an AIC value of 21976 and a BIC value of 22007 for the refined predictors. This again shows that the refined predictors were considerably better at predicting cognitive overload 5s after the DRT prompt.

**Fig. 14:**
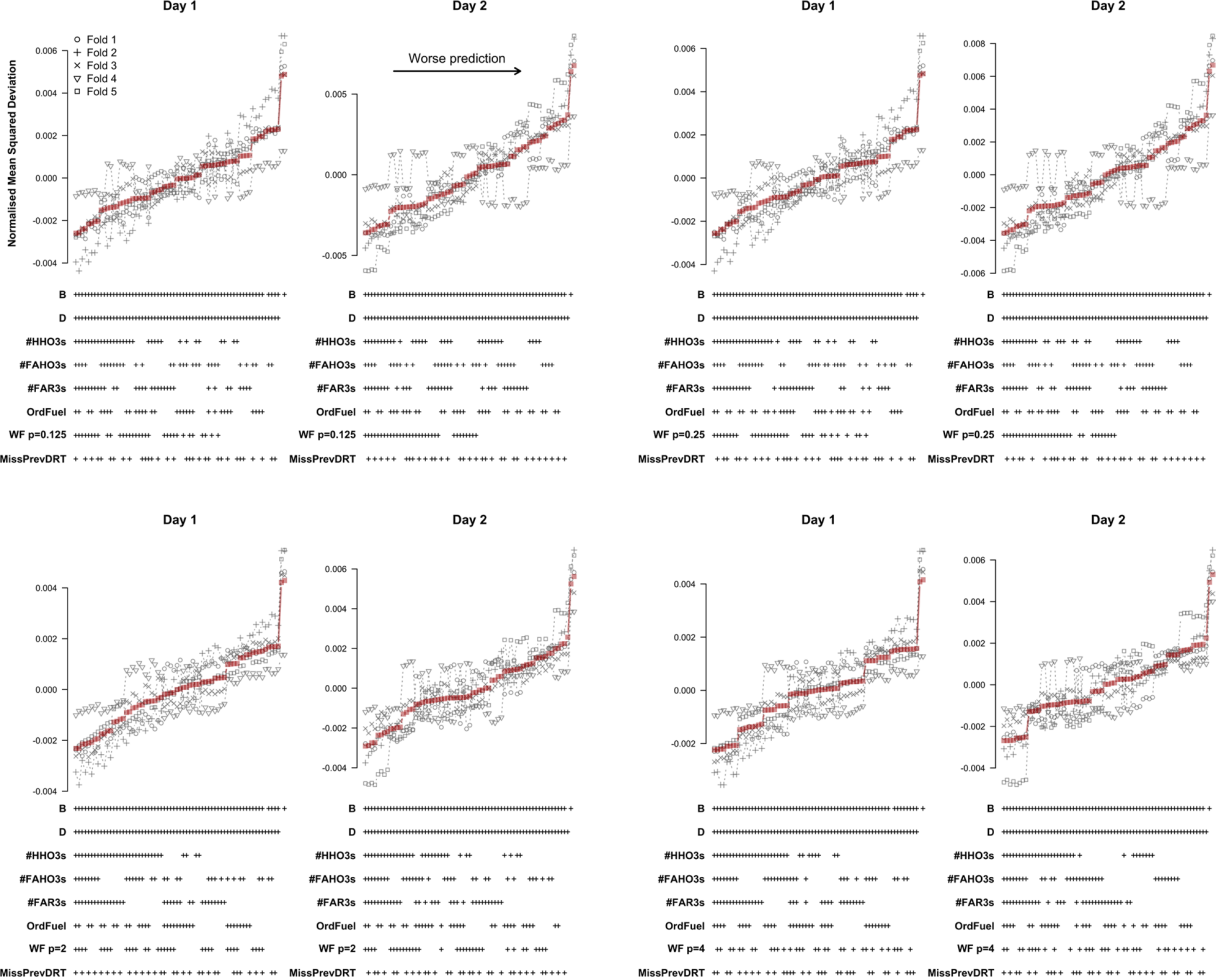
Cross-validation results for predicting log-RT from a TPM based on refined predictors with different exponents for the weighted average fuel-load predictor. Note that in order to make comparison easy the results in the top right panel repeat those in Fig. 11

**Fig. 15:**
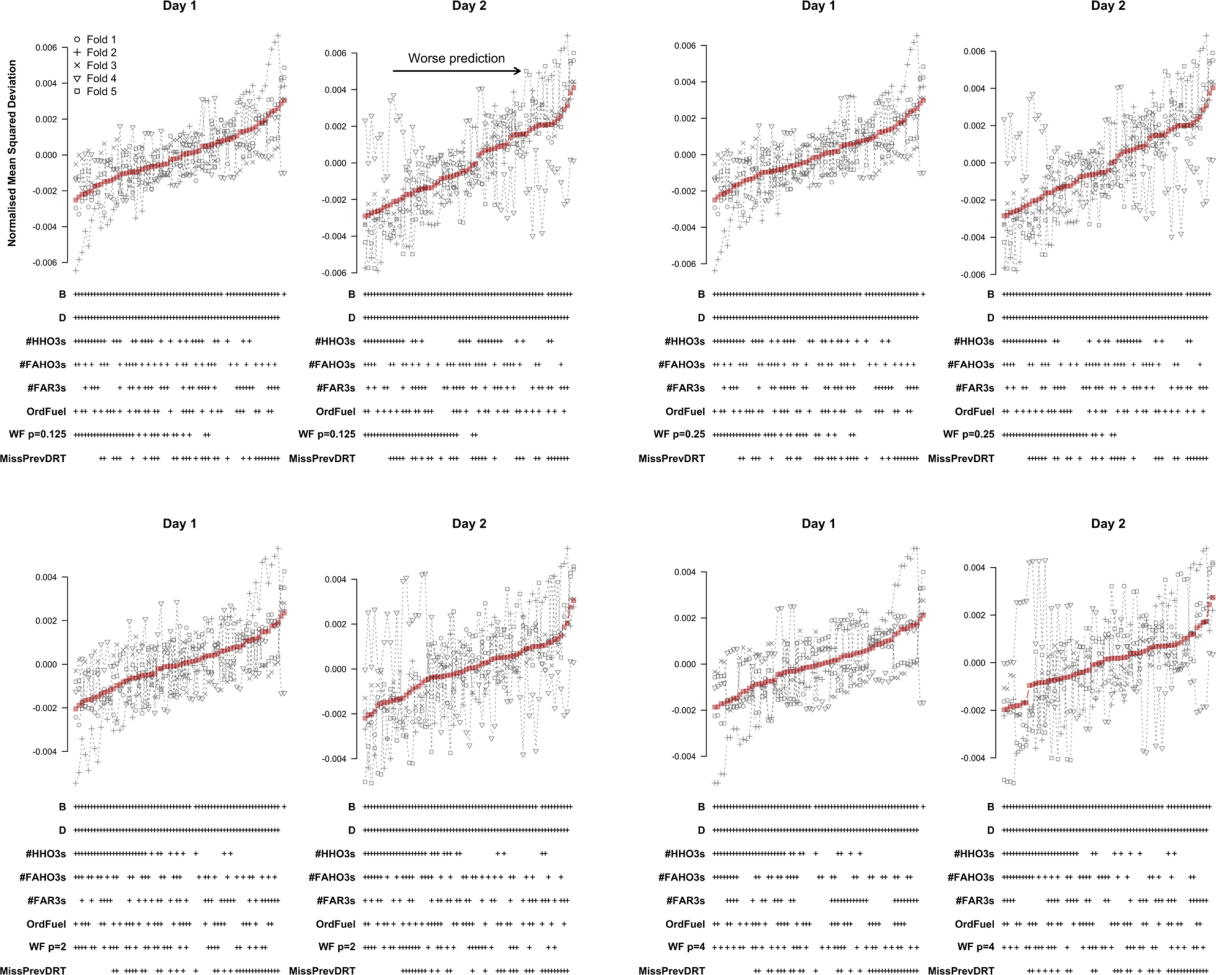
Cross-validation results for predicting log-RT from a LMM based on refined predictors with different exponents for the weighted average fuel-load predictor. Note that in order to make comparison easy the results in the top right panel repeat those in Fig. 11

One final question that remains is the influence our choice of the exponent of the Minkowski distance in Eq. (1) has on the predictive performance of WeightFuel. Our choice of $p=1/4$ was based on the assumption that participants attach higher importance to UAVs with lower fuel levels. According to this reasoning, computing the predictor with a different exponent $p<1$ should yield similar results, but using an exponent $p>1$ should yield worse predictive performance. To test this hypothesis, we repeated the cross-validation analysis for log-RTs with four different values of *p*: $p = 1/8$ (with corresponding WeightFuel predictor WF p = 1/8), $p=1/4$ (WF p = 1/4), $p=2$ (WF p = 2), and $p=4$ (WF p = 4). The results of the analysis based on TPM are shown in Fig. 14, and the results of the analysis based on LMMs are shown in Fig. 15.

Models in both figures are again presented in order of increasing average NMSD. As can be seen, for the two values of the exponent smaller than 1, the corresponding WeightFuel predictor is consistently included in the models with the lowest average NMSD, both in the TPMs and in the LMMs. For the two values of the exponent greater than 1, on the other hand, the corresponding WeightFuel predictor is included in several of the worst models.

## Discussion

Managing the effects of different levels of cognitive workload is important for both safety and productivity. Here we examined issues related to the ability of adaptive automation to undertake such management. We believe that for this approach to be most effective it is desirable to move beyond measuring past or present workload and instead *predict* future workload. For the first time to our knowledge, we explored the possibility of predicting real-time fluctuations in cognitive workload on short timescales (several seconds) based only on knowledge of factors related to the primary task and primary task performance. To do so, we had operators manage the refuelling of a fleet of unmanned ariel vehicles (UAVs), performing this task broken up into 24 two-minute blocks in each of two sessions occurring on different days. In order to develop and validate the predictive models we used the detection response task (DRT), an ISO standard (ISO 17488, 2016) methodology, to quantify cognitive workload in terms of responses to tactile stimulation occurring every 3-5 s. DRT performance is defined along two related dimensions, response time (RT) and omissions. We combined two linear mixed model analysis approaches, two-part models (TPMs) (Aitchison, 1955; Farewell et al., 2017) capable of modeling RT and omissions simultaneously and separate models of RT and omissions in order to identify potential predictors of cognitive workload. The separate models were required as current implementations of TPMs are limited in their ability to generate predictions for new cases based on estimates of the model coefficients from a separate dataset. Our aim was to determine if DRT RT and/or omission rates, both of which increase with cognitive workload, could be predicted from the recent history of the UAV task and the operator’s interactions with it. It is important to note that although our methodology required workload measurement to develop the predictive model, workload measurement is not required for an ongoing deployment, avoiding potential difficulties associated with user acceptance and interference with primary-task performance.

Preliminary analyses showed that over the two days of practice operators improved their performance in response to a payoff scheme, increasing occurrences of the two types of positive task events–checking for and replenishing fuel when it was low–and reducing occurrences of the three types of negative events–checking fuel and trying to refuel when the level was high, or allowing a UAV to run out of fuel so that it crashed. Both DRT omissions and RTs increased strongly with task difficulty, which was manipulated by varying the number of UAVs that had to be managed. In contrast, these measures decreased from day 1 to day 2, indicating that the cognitive demands of the task decreased with practice, although the difficulty effect remained clearly present on the second day. There was also a clear effect of blocks within sessions (i.e., time-on-task) with omissions increasing over the course of the sessions on both days. The same effect was seen on RT during day 2, but not on day 1, which may have been due to an underlying increase due to fatigue being mitigated by a decrease due to learning.

Our initial analysis assessed whether the occurrence of each of five task events in the last 3, 4, or 5 s predicted cognitive-workload fluctuations. We were not merely interested in assessing whether DRT performance differed significantly as a function of whether an event occurred or not. Instead, because of our focus on prediction, we used a much more demanding between-subject cross-validation test, which assessed whether including a task-event predictor in a model fit to DRT performance from one (training) subset of participants would enable that model to better predict DRT performance for another (test) set of participants. In all models, we included difficulty and time-on-task as predictors and then compared the effects of adding all possible combinations of the five events. This was repeated for five different folds—divisions of our participants into training and test sets—to assess the stability of predictions over individual differences.

The initial analysis confirmed that difficulty (“D”) and time-on-task (block; “B”) needed to be included. For the prediction of RT, the task events checking the fuel level, either successfully (i.e., when the UAV was ready for fueling; “HHO”) or unsuccessfully (“FAHO”), and attempting to refuel a UAV, either successfully (“HR”) of unsuccessfully (“FAR”), had predictive ability, with little variation in its performance across the three time intervals. For omissions, none of the task events had added predictive value. We took the effect of checking fuel as suggesting that, as workload increases, participants lost track of the UAV’s fuel level, and in order to compensate for this loss of situational awareness they needed to more frequently check their fuel levels.

We used the results of the initial analysis to create a refined set of six predictors, and focused on only the last 3 s as that provided slightly more consistent results in the initial analysis. Five predictors sought to better measure awareness about fuel load. The first three simply refined the most successful measures from the initial analysis, by counting the number of times fuel levels were checked successfully (“#HHO3s”), unsuccessfully (“#FAHO3s”), and the number of times UAVs were refuelled prematurely (“#FAR3s”). The fourth predictor was based on the rank of the fuel load of the last-checked UAV (from lowest to highest; “OrdFuel”), and was created with a similar rationale (i.e., that higher ranks corresponded to reduced situational awareness). The fifth was the average fuel levels of all UAVs (“WeightFuel”) with a higher weight given to lower loads because they were more indicative of an incipient UAV crash. Finally, we used a sixth predictor in the hope of specifically improving the poorer and more variable prediction performance for omissions that we found in the initial analysis, the occurrence of an omission for the previous DRT stimulus (“MissPrevDRT”). We speculated that omissions might occur in periods where participants forgot to respond to the DRT task, so an omission for the present DRT stimulus might be indexed by a previous omission. A previous omission might also predict a current omission occurring due to a prolonged period of high workload, in which case it would also predict RT, whereas forgetting arguably predicts a selective influence on omission rate.

The results of the second analysis were more encouraging for the prospect of developing real-time predictions. Once again difficulty (“D”) and time-on-task (block; “B”) were included in the best models for both RT and omissions. For RT there was also clear support for the refined version of the best predictors in the initial analysis, the number of successful fuel checks in the last 3 s (“#HHO3s”) and the number of premature refuelling attempts in the last 3 s (“#FAR3s”). Moreover, the weighted average fuel load (“WeightFuel”) was also strongly supported as a predictor. In contrast to the first analysis, the pattern of results was stable over folds. For omissions none of the refined predictors provided additional predictive information. Estimates of effect sizes suggested that a one standard deviation decrease in the weighted fuel level, and a one standard deviation increase in the number of successful fuel checks or the number of premature refuelling attempts had approximately the same effect on RT as an increase of 2 UAVs.

In a final analysis, we compared the ability of the best event-based predictors (HHO, FAHO, HR, FAR) and the best refined predictors (WeightFuel, #HHO3s, #FAR3s) in combination with difficulty (D) and time-on-task (B) to predict the worst game outcome, the number of exploded UAVs, during the 5-s time window following the DRT prompt. This analysis confirmed that the refined predictors were considerably better at predicting the worst game outcome that indicates complete cognitive overload and loss of situational awareness.

In summary, we identified task difficulty, time-on-task, weighted fuel load, and the number of successful fuel level checks and unsuccessful refuelling attempts as essential predictors of cognitive workload. We found that the refined predictors were able to account for the same sort of substantial change in cognitive workload as a consistent increase in the level of difficulty over several minutes, but where those changes occurred on a much shorter timescale. Thus, we were both able to confirm that fast fluctuations in task demands lead to correspondingly fast fluctuations in spare cognitive capacity and to identify a promising set of candidate measures that could be used to guide adaptive automation in real-world settings. We conclude by considering how these theoretical and practical results can form the basis of future research and applications.

### Limitations and future directions

A potential limitation of our results it that the link between events predictive of an increase in future workload and DRT performance may be mediated by an increase in the priority participants gave to the primary task, and hence a reduction in the capacity available for the DRT. This increase in priority might also result in an increase in the rate of user driven task events. Because we selected DRT responses to analyze based on proximity to task events [see Humphrey and Kramer (1994) for a similar methodology with ERPs] they would be likely to come from periods of higher primary task priority. Fortunately, although this could cause a range restriction that reduces predictive ability, it does not compromise the ability of the DRT to measure cognitive workload nor the fact that certain events are predictive of the DRT because they cause a change in cognitive workload associated with the primary task.

For omissions, the effect of failing to respond to a previous DRT stimulus had an even greater effect than the addition of two UAVs. In this case, the fact that this effect was selective to omissions suggests that it might index a somewhat different mechanism to the other predictors, perhaps related to goal neglect with respect to DRT responding. This might, in turn, be related to cognitive workload, but could also have other potential causes. The effect of the number of fuel checks was also selective, but for RT, suggesting that omissions and RT may have partially different drivers. Given that there was some divergence between RT and omission effects it would be desirable in future work using the DRT to attempt to develop an analysis that simultaneously accounted for both measures in a more theoretically principled way than our descriptive two-part models. In a future investigation we plan to use the reduced set of candidate predictors in combination with evidence-accumulation models (e.g., Tillman et al., 2017) to simultaneously model RT and omission data, in order to assess whether this yields better real-time prediction.

We used between-subject cross-validation here because we were interested in determining if primary-task characteristics could be predictive for all participants, and hence whether it is viable to develop a general predictive model for a given task that can be applied to new users without needing to collect workload measurement for them. Although we did find this to be the case to a large degree, there were also clear individual differences. It may, therefore, be illuminating to also perform variable-selection based on within-subject cross-validation. Performance is likely to be better, but more interesting would be the possibility of individual differences in the best predictors. If they occurred then it would suggest that it would be useful to calibrate predictive models for individual operators not only in a quantitative sense, but also in terms of the predictors used. Our results also suggest that predictive models might need to take account of changes within users over time, such as those associated with skill acquisition. In order to tune the predicative model in an ongoing manner, it might be useful to consider a role for an embedded secondary task (Raby and Wickens 1994), a relatively frequent but low priority response that is a legitimate part of the operator’s duties.

More effective prediction might also be obtained based on different or additional workload measures. This might be particularly desirable where successful performance in the primary task depends on multiple cognitive resources determining workload, or on factors other than cognitive workload. One possibility is to use a set of different secondary-task measures that address the different relevant cognitive resources (Schlegel et al., 1986). Another possibility is to use either one or more physiological measures either with or without secondary-task measures. Developing predictive models based on physiological measures may be particularly appropriate where it is desirable for adaptive automation to take account of other factors such as stress or fatigue, and because of their potential to be less obtrusive physiological measures may have utility in ongoing tuning of predictive models.

Although our second variable-selection analysis explored only a relatively small number of predictors, so that we cannot rule out that other better predictors exist, it does at least confirm that real-time prediction based on factors related to the primary task appears viable. These results also suggest that, at least for the present task, there are relatively rapid fluctuations of cognitive workload that have a sufficiently substantial magnitude to make them a worthwhile target for adaptive automation. The same methodology used here—benchmarking the magnitude of short-term fluctuations against difficulty manipulations that are clearly related to cognitive workload—can be used to establish if fluctuations in other tasks are similarly large.

The results of the refined variable-selection analysis are also salutatory for applying our methods more generally. They suggest that at least one route to successful prediction relies on quantifying factors related to the mental states that operators require to enable successful control, which in the case of our task was situational awareness about UAV fuel levels. As maintenance of control is a characteristic common to many tasks, this suggests potential for generalizing the current approach to a broad range of other tasks. To do so successfully, it seems likely that researchers should consider as predictors primary task characteristics that are related to attaining the task’s goals. The results of such analyses could also have utility beyond guiding adaptive automation, by identifying aspects of task design that might be improved to reduce cognitive workload.

To sum up, the aim of the present work was to develop a method for predicting short-term fluctuations in cognitive workload in a complex monitoring task. Using a cross-validation approach with performance on a secondary DRT task as criterion, we identified primary task events that were predictive of users’ cognitive workload in a 3 to 5-s time window. The success of this approach in a relatively complex simulated task environment suggests that it might also be successfully generalized to complex real-world domains. A particularly attractive feature of our method is its reliance on primary task events, which minimizes interference with ongoing task performance and opens up the possibility of noninvasive real-time prediction of rapid workload fluctuations.

## Data Availability

All data, analysis scripts, and supplementary materials are available at https://osf.io/wqzsg/

## Footnotes

1 The log odds of an omission are computed as $\log (p/(1-p))$, where *p* is the probability of an omission. Although the mapping is not linear in *p*, it is strictly increasing, which means that larger beta weights correspond to a higher probability of an omission.

## References

[CR1] Abdelrahman Y, Velloso E, Dingler T, Schmidt A, Vetere F (2017). Cognitive heat. Proceedings of the ACM on Interactive, Mobile, Wearable and Ubiquitous Technologies.

[CR2] Aho K, Berryberry D, Peterson T (2014). Model selection for ecologists: The worldviews of AIC and BIC. Ecology.

[CR3] Aitchison OO (1955). On the distribution of a positive random variable having a discrete probability mass at the origin. Journal of the American Statistical Association.

[CR4] Akaike H, Petrov BN, Caski S (1973). Information theory and an extension of the maximum likelihood principle. Proceedings of the second international symposium on information theory.

[CR5] Allison BZ, Polich J (2008). Workload assessment of computer gaming using a single-stimulus event-related potential paradigm. Biological Psychology.

[CR6] Aricò P, Borghini G, Di Flumeri G, Colosimo A, Bonelli S, Golfetti A, Pozzi S, Imbert J-P, Granger G, Benhacene R, Babiloni F (2016). Adaptive automation triggered by EEG-based mental workload index: A passive brain-computer interface application in realistic air traffic control environment. Frontiers in Human Neuroscience.

[CR7] Ayaz H, Shewokis PA, Bunce S, Izzetoglu K, Willems B, Onaral B (2012). Optical brain monitoring for operator training and mental workload assessment. NeuroImage.

[CR8] Bengler K, Kohlmann M, Lange C (2012). Assessment of cognitive workload of in-vehicle systems using a visual peripheral and tactile detection task setting. Work: A Journal of Prevention, Assessment and Rehabilitation.

[CR9] Borghini G, Astolfi L, Vecchiato G, Mattia D, Babiloni F (2014). Measuring neurophysiological signals in aircraft pilots and car drivers for the assessment of mental workload, fatigue and drowsiness. Neuroscience & Biobehavioral Reviews.

[CR10] Bruyas, M. P., & Dumont, L. (2012). Sensitivity of Detection Response Task (DRT) to the driving demand and task difficulty. In: *Proceedings of the seventh international driving symposium on human factors in driver assessment, training, and vehicle design* (pp. 64–70). University of Iowa.

[CR11] Burnham KP, Anderson DR, Huyvaert KP (2011). AIC model selection and multimodel inference in behavioral ecology: Some background, observations, and comparisons. Behavioral Ecology and Sociobiology.

[CR12] Byrne EA, Parasuraman R (1996). Psychophysiology and adaptive automation. Biological Psychology.

[CR13] Castro, S., Strayer, D., Matzke, D., & Heathcote, A. (2019). Cognitive workload measurement and modelling under divided attention. *Journal of Experimental Psychology: Human Perception and Performance, 45(6), 826–839.* 10.1037/xhp0000638 30998070

[CR14] Chen L-L, Zhao Y, Ye P-F, Zhang J, Zou J-z (2017). Detecting driving stress in physiological signals based on multimodal feature analysis and kernel classifiers. Expert Systems with Applications.

[CR15] De Massari D, Pacheco D, Malekshahi R, Betella A, Verschure PFMJ, Birbaumer N, Caria A (2014). Fast mental states decoding in mixed reality. Frontiers in Behavioral Neuroscience.

[CR16] Defayolle M, Dinand J, Gentil M, Singleton W, Fox J, Whitfield D (1971). Averaged evoked potentials in relation to attitude, mental load and intelligence. Measurement of man at work.

[CR17] Duchowski, A. T., Krejtz, K., Krejtz, I., Biele, C., Niedzielska, A., Kiefer, P., Raubal, M., & Giannopoulos, I. (2018). The index of pupillary activity: measuring cognitive load vis-à-vis task difficulty with pupil oscillation. In *Proceedings of the 2018 CHI conference on human factors in computing systems*.

[CR18] Farewell VT, Long DL, Tom BDM, Yiu S, Su L (2017). Two-part and related models for longitudinal data. Annual Review of Statistics and Its Applications.

[CR19] Gevins A, Leong H, Du R, Smith ME, Le J, DuRousseau D, Zhang J, Libove J (1995). Towards measurement of brain function in operational environments. Biological Psychology.

[CR20] Gevins AS, Smith ME, Leong H, McEvoy L, Whitfield S, Du R, Rush G (1998). Monitoring working memory load during computer-based tasks with EEG pattern recognition methods. Human Factors: The Journal of the Human Factors and Ergonomics Society.

[CR21] Gomer F, Morael J, Kraiss K (1981). Physiological monitoring and the concept of adaptive systems. Manned systems design.

[CR22] Gopher D, Donchin E, Boff KR, Kaufman L, Thomas JP (1986). Workload: An examination of the concept. Handbook of perception and human performance, Vol. 2. Cognitive processes and performance.

[CR23] Groll-Knapp E, Singleton W, Fox J, Whitfield D (1971). Evoked potentials and behavior. Measurement of man at work.

[CR24] Harbluk, J. L., Burns, P. C., Hernandez, S., Tam, J., & Glazduri, V. (2012). Detection response tasks: Using remote, headmounted and Tactile signals to assess cognitive demand while driving. In *Proceedings of the seventh international driving symposium on human factors in driver assessment, training, and vehicle design* (pp. 78–84). University of Iowa.

[CR25] Hart SG (2006). Nasa-Task Load Index (NASA-TLX); 20 years later. Proceedings of the Human Factors and Ergonomics Society Annual Meeting.

[CR26] Hastie T, Tibshirani R, Friedman J (2009). The elements of statistical learning.

[CR27] Hawkins GE, Mittner M, Boekel W, Heathcote A, Forstmann BU (2015). Toward a model-based cognitive neuroscience of mind wandering. Neuroscience.

[CR28] Heine T, Lenis G, Reichensperger P, Beran T, Doessel O, Deml B (2017). Electrocardiographic features for the measurement of drivers’ mental workload. Applied Ergonomics.

[CR29] Humphrey DG, Kramer AF (1994). Toward a psychophysiological assessment of dynamic changes in mental workload. Human Factors.

[CR30] ISO 17488. (2013). Road Vehicles—Transport information and control systems—Detection Response Task (DRT) for assessing selective attention in driving.

[CR31] Kahnamen D (1973). Attention and effort.

[CR32] Koul A, Becchio C, Cavallo A (2018). Cross-validation approaches for replicability in psychology. Frontiers in Psychology.

[CR33] Kramer AF, Trejo LJ, Humphrey D (1995). Assessment of mental workload with task-irrelevant auditory probes. Biological Psychology.

[CR34] Le AS, Aoki H, Murase F, Ishida K (2018). A novel method for classifying driver mental workload under naturalistic conditions with information from near-infrared spectroscopy. Frontiers in Human Neuroscience.

[CR35] Liang B, Lin Y (2018). Using physiological and behavioral measurements in a picture-based road hazard perception experiment to classify risky and safe drivers. Transportation Research Part F: Psychology and Behaviour.

[CR36] Lohani M, Payne BR, Strayer DL (2019). A review of psychophysiological measures to assess cognitive states in real-world driving. Frontiers in Human Neuroscience.

[CR37] Medeiros-Ward N, Cooper JM, Strayer DL (2014). Hierarchical control and driving. Journal of Experimental Psychology: General.

[CR38] Mijović P, Ković V, De Vos M, Mačužić I, Todorović P, Jeremić B, Gligorijević I (2017). Towards continuous and real-time attention monitoring at work: Reaction time versus brain response. Ergonomics.

[CR39] Murata A (2005). An Attempt to Evaluate Mental Workload Using Wavelet Transform of EEG. Human Factors: The Journal of the Human Factors and Ergonomics Society.

[CR40] Palada, H., Neal, A., Strayer, D., Ballard, T., & Heathcote, A. (in press). Competing for cognitive resources: Measuring workload in a time pressured dual-task environment. *Journal of Experimental Psychology: Human Perception and Performance*. 10.1037/xhp0000672 31282694

[CR41] Pashler H (1984). Processing stages in overlapping tasks: Evidence for a central bottleneck. Journal of Experimental Psychology: Human Perception and Performance.

[CR42] Pashler H (1994). Dual-task interference in simple tasks: Data and theory. Psychological Bulletin.

[CR43] Pergher V, Wittevrongel B, Tournoy J, Schoenmakers B, Van Hulle MM (2019). Mental workload of young and older adults gauged with ERPs and spectral power during N-Back task performance. Biological Psychology.

[CR44] Pinheiro CJ, Bates DM (2000). Mixed-effects Models in S and S-Plus.

[CR45] Pope AT, Bogart EH, Bartolome DS (1995). Biocybernetic system evaluates indices of operator engagement in automated task. Biological Psychology.

[CR46] Posner MI (1978). Chronometric explorations of mind.

[CR47] Posner MI, Boies SJ (1971). Components of attention. Psychological Review.

[CR48] Prinzel LJ, Freeman FG, Human MS (2003). 2003: Effects of a psychophysiological system for adaptive automation on performance, workload, and the event-related potential P300 component. Human Factors.

[CR49] Prinzel LJ, Freeman FG, Scerbo MW, Mikulka PJ, Pope AT (2000). A closed-loop system for examining psychophysiological measures for adaptive task allocation. The International Journal of Aviation Psychology.

[CR50] Quandt, J. (2017). Automatic vehicle control systems [Investigation No. PE 16–007]. Technical report, US Department of Transportation: National Highway Traffic Safety Administration.

[CR51] Raby M, Wickens CD (1994). Strategic workload management and decision biases in aviation. The International Journal of Aviation Psychology.

[CR52] Rajdev P, Thorsley D, Rajaraman S, Rupp TL, Wesensten NJ, Balkin TJ, Reifman J (2013). A unified mathematical model to quantify performance impairment for both chronic sleep restriction and total sleep deprivation. Journal of Theoretical Biology.

[CR53] Ratcliff R (1978). A theory of memory retrieval. Psychological Review.

[CR54] Rizopoulos, D. (2020). Generalized Linear Mixed Models using Adaptive Gaussian Quadrature [R package] . https://CRAN.R-project.org/package=GLMMadaptive.

[CR55] Schlegel RE, Gilliland K, Schlegel B (1986). Development of the criterion task set performance data base. Proceedings of the meeting of the 30th annual Human Factors Society.

[CR56] Schwarz G (1978). Estimating the dimension of a model. The Annals of Statistiscs.

[CR57] Sem-Jacobson C (1981). Brain/computer communications to reduce human error: A perspective. Aviation, Space and Environmental Medicine.

[CR58] Sirevaag EJ, Kramer AF, Wickens CD, Reisweber M, Strayer DL, Grenell JH (1993). Assessment of pilot performance and mental workload in rotary wing aircraft. Ergonomics.

[CR59] Smith ME, Gevins AS, Brown H, Karnik A, Du R (2001). Monitoring task loading with multivariate EEG measures during complex forms of human-computer interaction. Human Factors: The Journal of the Human Factors and Ergonomics Society.

[CR60] Strayer, D. L., Biondi, F., & Cooper, J. M. (2017). Dynamic workload fluctuations in driver/non-driver conversational dyads. In D. V. McGehee, J. D. Lee, & M. Rizzo (Eds.), *Driving assessment 2017: international symposium on human factors in driver assessment, training, and vehicle design* (pp. 362–367). University of Iowa, Public Policy Center.

[CR61] Strayer DL, Cooper JM, Turrill J, Coleman JR, Hopman RJ (2016). Talking to your car can drive you to distraction. Cognitive Research: Principles and Implications..

[CR62] Strayer DL, Cooper JM, Turrill J, Coleman JR, Hopman RJ (2017). The smartphone and the driver’s cognitive workload: A comparison of Apple, Google, and Microsoft’s intelligent personal assistants. Canadian Journal of Experimental Psychology.

[CR63] Strayer DL, Turrill J, Cooper JM, Coleman JR, Medeiros-Ward N, Biondi F (2015). Assessing cognitive distraction in the automobile. Human Factors: The Journal of the Human Factors and Ergonomics Society.

[CR64] Teh E, Jamson S, Carsten O, Jamson H (2014). Temporal fluctuations in driving demand: The effect of traffic complexity on subjective measures of workload and driving performance. Transportation Research Part F: Traffic Psychology and Behaviour.

[CR65] Tillman G, Strayer D, Eidels A, Heathcote A (2017). Modeling cognitive load effects of conversation between a passenger and driver. Attentation, Perception, & Psychophysics.

[CR66] Unity Technologies: Unity® (version 2017.3.1f1) [Computer software] (2017). http://unity3d.com.

[CR67] Visnovcova Z, Mestanik M, Gala M, Mestanikova A, Tonhajzerova I (2016). The complexity of electrodermal activity is altered in mental cognitive stressors. Computers in Biology and Medicine.

[CR68] Welch J (1898). On the measurement of mental activity through muscular activity and the determination of a constant of attention. American Journal of Physiology.

[CR69] Welford AT (1958). Perception and Communication.

[CR70] Welford AT (1968). Fundamentals of Skill.

[CR71] Welford AT, Time Reaction (1981). The single-channel hypothesis. Welford AT.

[CR72] Wickelgren WA (1977). Speed-accuracy tradeoff and information processing dynamics. Acta Psychologica.

[CR73] Wickens CD, Parasuraman R, Davies DR (1984). Processing resources in attention. Varieties of attention.

[CR74] Wickens CD, Hollands JG, Banbury S, Parasuraman R (2013). Engineering psychology and human performance.

[CR75] Yarkoni T, Westfall J (2017). Choosing prediction over explanation in psychology: Lessons from machine learning. Perspectives on Psychological Science.

