# Correction to: Real-time prediction of short-timescale fluctuations in cognitive workload

**DOI:** 10.1186/s41235-021-00328-8

**Published:** 2021-09-23

**Authors:** Udo Boehm, Dora Matzke, Matthew Gretton, Spencer Castro, Joel Cooper, Michael Skinner, David Strayer, Andrew Heathcote

**Affiliations:** 1 Department of Psychology, University of Amsterdam, PO Box 15906, 1001 NK Amsterdam, The Netherlands; 2 Department of Psychology, University of Tasmania, Sandy Bay, Australia; 3 Department of Psychology, University of Utah, Utah, USA; 4 Aerospace Division, Defence Science and Technology Group, Melbourne, Australia

## Correction to: Cogn. Research (2021) 6:30 10.1186/s41235-021-00289-y

The original article (Boehm 2021) contained a typesetting error whereby the columns of text in Page 11 of the article’s PDF were erroneously swapped. This error was not the fault of the author but instead a technical error incorporated during production of the article.

The typesetting in the original article has since been corrected.
